# Public Health Impact of Paxlovid as Treatment for COVID-19, United States

**DOI:** 10.3201/eid3002.230835

**Published:** 2024-02

**Authors:** Yuan Bai, Zhanwei Du, Lin Wang, Eric H.Y. Lau, Isaac Chun-Hai Fung, Petter Holme, Benjamin J. Cowling, Alison P. Galvani, Robert M. Krug, Lauren Ancel Meyers

**Affiliations:** The University of Hong Kong, Hong Kong (Y. Bai, Z. Du, E.H.Y. Lau, B.J. Cowling);; Hong Kong Science and Technology Park, Hong Kong, China (Y. Bai, Z. Du, E.H.Y. Lau, B.J. Cowling);; University of Cambridge, Cambridge, UK (L. Wang);; Deakin University, Burwood, Victoria, Australia (E.H.Y. Lau);; Georgia Southern University, Statesboro, Georgia, USA (I. C.-H. Fung);; Aalto University, Espoo, Finland (P. Holme); Kobe University, Kobe, Japan (P. Holme);; Yale School of Public Health, New Haven, Connecticut, USA (A.P. Galvani);; University of Texas at Austin, Austin, Texas, USA (R.M. Krug, L.A. Meyers);; Santa Fe Institute, Santa Fe, New Mexico, USA (L.A. Meyers)

**Keywords:** COVID-19, Paxlovid, coronavirus disease, SARS-CoV-2, severe acute respiratory syndrome coronavirus 2, viruses, respiratory infections, zoonoses, SARS-CoV-2 transmission, public health impact, treatment, mathematical model, United States

## Abstract

We evaluated the population-level benefits of expanding treatment with the antiviral drug Paxlovid (nirmatrelvir/ritonavir) in the United States for SARS-CoV-2 Omicron variant infections. Using a multiscale mathematical model, we found that treating 20% of symptomatic case-patients with Paxlovid over a period of 300 days beginning in January 2022 resulted in life and cost savings. In a low-transmission scenario (effective reproduction number of 1.2), this approach could avert 0.28 million (95% CI 0.03–0.59 million) hospitalizations and save US $56.95 billion (95% CI US $2.62–$122.63 billion). In a higher transmission scenario (effective reproduction number of 3), the benefits increase, potentially preventing 0.85 million (95% CI 0.36–1.38 million) hospitalizations and saving US $170.17 billion (95% CI US $60.49–$286.14 billion). Our findings suggest that timely and widespread use of Paxlovid could be an effective and economical approach to mitigate the effects of COVID-19.

Antiviral drugs can substantially reduce illness and deaths from human infections. For example, antiretroviral therapy has prevented millions of HIV/AIDS deaths globally since the late 1980s (*1*). During the 2009 influenza A(H1N1) pandemic, oseltamivir was widely administered in the United States (28.4 prescriptions/1,000 persons) (*2*); rapid treatment after symptom onset reduced the risk for hospitalization by an estimated 63% (95% CI 17%–81%) (*3*). The reduction in viral load might reduce the risk for onward transmission while accelerating recovery. A counterfactual analysis suggests that treating even 10% of infected patients with baloxavir shortly after symptom onset would have prevented millions of infections and thousands of deaths in the United States during the severe 2017–18 influenza season (*4*). A fast-acting SARS-CoV-2 antiviral could similarly be deployed to curtail transmission on a population scale and directly save lives (*5*).

Paxlovid (Pfizer, https://www.pfizer.com), which received Food and Drug Administration Emergency Use Authorization on December 22, 2021, for treating SARS-CoV-2 infections in persons >12 years of age, combines 2 different antiviral agents, nirmatrelvir and ritonavir. Treating symptomatic COVID-19 patients with Paxlovid reduces hospitalization risks by an estimated 0.59 (95% CI 0.48–0.71) for adults 18–49 years of age, 0.40 (95% CI 0.34–0.48) for adults 50–64 years of age, and 0.53 (95% CI 0.48–0.58) for adults >64 years of age (*6*). Paxlovid has proven effective against the Omicron variant (*7*). In January 2022, the United States ordered 20 million courses of Paxlovid to be delivered within 9 months (*8*).

In this study, we analyzed the population-level benefits of expanding the clinical use of Paxlovid to treat COVID-19. By fitting a within-host model of viral replication to viral titer data from >2,000 COVID-19 patients, we provide early estimates for the efficacy of Paxlovid in curtailing viral load, depending on the timing of treatment after infection. Then, using a population-level SARS-CoV-2 transmission model, we estimated the effects of Paxlovid-based interventions on reducing the healthcare and economic burden of future COVID-19 epidemics. Specifically, we estimated the number of cases, hospitalizations, and deaths, as well as healthcare costs averted under a range of transmission scenarios, in which we vary both the between-individual transmission rate of the virus and the proportion of case-patients who receive rapid treatment with Paxlovid. This 2-level analytic framework can broadly support the rapid evaluation of antiviral-based mitigation strategies against COVID-19 and other respiratory viruses (*4*).

## Materials and Methods

### Within-Host Model of SARS-CoV-2 Replication Dynamics

We simulated SARS-CoV-2 virus kinetics in an infected person and the effect of Paxlovid treatment on viral growth using a standard target-cell limited virus kinetic model that tracks the number of uninfected cells, infected cells, and free viral particles (*9*,*10*) (Appendix). We used individual patient viral load data from a Paxlovid clinical trial data to estimate the 5 key parameters of the model: the infection rate of susceptible cells (*b*), the rate at which infected cells die , the rate at which active viruses were cleared (*c*), the virus production rate (*p*), and the efficacy of Paxlovid at suppressing viral replication (). Specifically, we used a stochastic approximation expectation-maximization algorithm to fit the model to 14-day viral titer data from 1,126 infected adults treated with a placebo and 1,120 infected adults treated with Paxlovid during a clinical trial in late 2021 (*11*) (Appendix).

### Modeling the Infectiousness of Treated and Untreated Cases

On the basis of previous studies (*12*,*13*), we assumed that a person’s infectiousness is logarithmically related to their viral titer (Appendix). In this transmission model, we assumed that the daily infectiousness of a case-patient depends on whether they received treatment and, if so, the time at which treatment was initiated after symptom onset. To estimate the daily infectiousness of a given untreated or treated case-patient, we first used the within-host model to simulate the viral load on each day of the infection and set the viral load to zero when the estimated value dropped below the detection threshold of 100 (*14*). We then used a logarithmic equation (Appendix) to estimate the corresponding daily infectiousness.

### Modeling Population-Level SARS-CoV-2 Transmission Dynamics and Effects of Antiviral Treatment

We developed a stochastic individual-based network model of SARS-CoV-2 transmission dynamics in which susceptible persons can be infected by infected contacts (Appendix Figure 1). The underlying contact network included 9,961 persons living in 5,000 households with sociodemographic characteristics provided in the 2017 National Household Travel Survey (*15*,*16*) (Appendix).

At every time point, each person was in one of 11 possible states: unvaccinated susceptible (S_U_), vaccinated susceptible (S_V_), exposed (E), presymptomatic (P), symptomatic infectious before becoming eligible for Paxlovid treatment (Y), symptomatic treated (Y_T_), symptomatic untreated (Y_U_), asymptomatic infectious (A), recovered (R), hospitalized (H), or deceased (D). We assumed that hospitalized patients were isolated and not able to infect others. Upon infection, a susceptible person progresses to the exposed state and then to either the presymptomatic state (probability ψ) or asymptomatic state (probability 1 − ψ). Asymptomatic case-patients recover without experiencing symptoms or seeking treatment. Presymptomatic case-patients progress to the symptomatic state at a rate ω, where they might be hospitalized according to published age-specific infection hospitalization rates (*h_a_*) and eventually recover or die from the infection, according to age-specific infection fatality rates (*μ_a_*). A fraction ρ of symptomatic case-patients receive Paxlovid, initiated an average of 3 days after symptom onset, which is assumed to reduce the risk for hospitalization (*φ_a_*), as well as the infectiousness of the person. The infectiousness of a case-patient depends on the timing of Paxlovid administration after infection, according to the daily infectiousness curves described in the previous section. Vaccinated persons initially have vaccine-derived immunity against infection *ω_B_*, symptomatic disease *ψ_B_*, and death *θ_B_*, which wanes gradually after vaccination. Similarly, recovered persons initially have infection-derived immunity against reinfection *ω_N_*, symptomatic disease *ψ_N_*, and death *θ_B_*, which wanes more slowly than vaccine-derived immunity. Persons who are vaccinated and previously infected are assumed to have the higher of the 2 levels of immunity (i.e., infection-acquired vs. immune-acquired) (Table 1; Appendix Tables 1, 2). 

**Table 1:** Between-host parameter estimates used in study of public health impact of Paxlovid in treatment of COVID-19, United States*

Key parameter	Estimated value
Symptomatic proportion, % (ψ)	75
Transition rate out of exposed state (d^–1^) ()	1/3
Time lag between infection and recovery in days for asymptomatic patients (d^–1^) (*γ_A_*)	1/9
Time lag between symptom onset and recovery in days for symptomatic patients (d^–1^) (γ*_T_*)	1/4
Transition rate from the presymptomatic to the symptomatic stage (d^–1^) (ω)	1/2
Age-specific efficacy of Paxlovid in reducing the hospitalization rate, y (*φ_a_*)	
0–4	0.59 (95% CI 0.48–0.71)
5–17	0.59 (95% CI 0.48–0.71)
18–49	0.59 (95% CI 0.48–0.71)
50–64	0.40 (95% CI 0.34–0.48)
>65	0.53 (95% CI 0.48–0.58)
Life expectancy, y, for age group *a*, adjusted assuming a 3% yearly discount rate (*λ_a_*)	
0–4	30.3
5–17	29.3
18–49	25.8
50–64	1837
>65	12.9

*We use the between-host model to project population-level impacts of Paxlovid treatment. Key parameter values used in the model are listed below, with more details in Appendix Table 1).

### Antiviral Treatment and Transmission Scenarios

We analyzed 24 different scenarios, each with an effective reproduction number (R_t_) (1.2, 1.5, 1.7, 2, 3, or 5) and Paxlovid treatment rate (20%, 50%, 80%, or 100%). For each scenario (*s*), we compared 4 variations of the antiviral strategy: no treatment (i.e., treatment rate set to zero); treatment with Paxlovid at the given treatment rate; treatment with a hypothetical antiviral that reduces infectiousness with the same efficacy as Paxlovid but does not reduce severity; and treatment with a hypothetical antiviral that reduces severity with the same efficacy as Paxlovid but does not reduce infectiousness. The last 2 variations enabled us to separate the direct therapeutic benefits from the indirect transmission-blocking benefits of Paxlovid. To estimate the health and economic costs associated with each scenario, we ran 100 stochastic simulations of each of the 4 strategy variations and calculated the mean and 95% CI across simulations of the years of life lost (YLL) averted and monetary costs attributable to Paxlovid treatment.

### Estimating YLL Averted and Monetary Costs

For each set of stochastic simulations, we estimated YLL averted for each antiviral strategy τ by comparing it to the no treatment strategy (Appendix). The willingness to pay per YLL averted is the maximum price a society is willing to pay to prevent the loss of 1 year of life. Health economists have inferred from healthcare expenditure that the United States is willing to pay US $100,000 per quality-adjusted life-year (*17*), of which YLL is 1 component. For a given willingness to pay for a YLL averted (*θ*), we calculated the net monetary benefit (NMB) of each strategy (Appendix).

### Sensitivity Analyses and Model Validation

**Figure 1:**
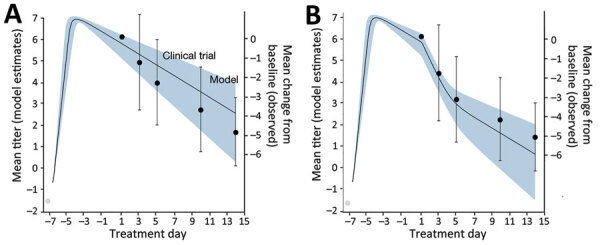
Estimated and observed viral load following treatment with placebo (A) or Paxlovid (B) in large-scale campaign treating COVID-19, United States. The left y-axes, black lines, and blue shading indicate the means and 95% CI of SARS-CoV-2 viral load (RNA log_10_ copies/mL) as estimated by the fitted within-host model. The right y-axes, black dots, and error bars indicate the means and 95% CI of the decrease in viral load since the initiation of treatment as reported in a clinical trial in which 1,126 patients received a placebo and 1,120 patients received Paxlovid during July 16–December 9, 2021 (*11*). Day one corresponds to the initiation of treatment. Gray circles denote the assumed initial viral load upon infection (V_0_) corresponding to 1 infectious virus particle in the upper respiratory tract (*18*).

We assessed the robustness of the results with respect to the relationship between infectiousness and viral load by investigating 3 alternative functions (i.e., sigmoid, log-proportional, and step) (Appendix Tables 5, 6). To validate our within-host viral replication mode, we compared model-estimated mean viral load trajectories for untreated and treated case-patients to corresponding clinical trial data for patients receiving placebo or Paxlovid treatment (*1*). We found that the observed mean decreases in viral load fall within the estimated 95% CI and vice versa (Figure 1; Appendix Figure 3).

To validate our transmission dynamic model, we compared model projections to observed incidence data during the early 2022 and late 2022 Omicron waves in the United States (Appendix Figure 2). For each of these waves, we fitted the model to reported case data to estimate the initial R_t_ and then simulated the expected reported infections, assuming a 25% case-reporting rate (*7*).

## Results

By fitting the within-host model to the mean viral load dynamics reported from a clinical trial (Table 2; Figure 1), we estimated that the rate at which viral particles infect susceptible cells (b) is 3.92 (95% CI 2.82–5.38) × 10^−6^ mL/copies/day), the clearance rate for infected cells (δ) is 0.62 (95% CI 0.42–0.92) per day, the rate at which infected cells release virus (p) is 3.19 (95% CI 2.35–4.35) copies/mL/day/cell, and the rate at which free virus particles are cleared (c) is 2.21 (95% CI 2.10–2.33) per day. Treatment with Paxlovid is estimated to repress viral replication by 99.37% (95% CI 99.17%–99.52%) per day.

**Table 2:** Within-host parameter estimates used in study of public health impact of Paxlovid in treatment of COVID-19, United States*

Parameter	Mean (95% CI)
Cell infection rate in 10^−6^ mL/copies/day (b)	3.92 (2.82–5.38)
Infected cell death rate per day ()	0.62 (0.42–0.92)
Virus production rate in copies/mL/day/cell (*p*)	3.19 (2.35–4.35)
Virus death rate per day (c)	2.21 (2.10–2.33)
Antiviral efficacy ()	0.9937 (0.9917–0.9952)

*We fit the within-host model to the mean viral load dynamics reported from a clinical trial involving 2,246 infected adults treated with either Paxlovid or a placebo (*11*) using nonlinear mixed-effects model method (*19*). This method allows between-subject variability to improve the precision and accuracy of estimates (*20*). Values are means and 95% CI of parameter values in population, assuming that antiviral efficacy follows logit-normal distribution, and all other individual parameters follow log-normal distributions.

**Figure 2:**
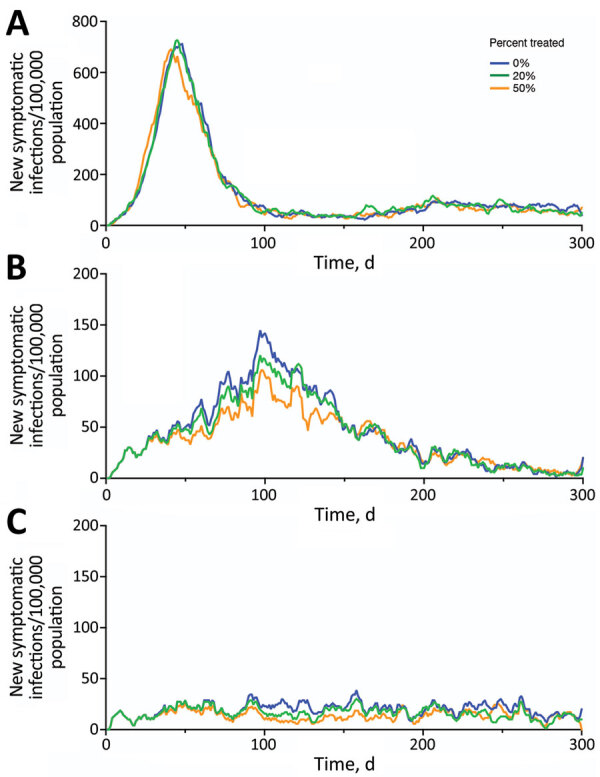
Projected symptomatic SARS-CoV-2 infections over 300 days in the United States across a range of transmission and Paxlovid treatment scenarios. Estimated incidence of symptomatic SARS-CoV-2 infections are shown assuming an effective reproduction number of 3.0 (A), 1.7 (B), or 1.2 (C). Colors correspond to 3 different treatment scenarios: 0% (blue), 20% (green), or 50% (orange) of symptomatic cases received a 5-day Paxlovid regimen initiated within 3 days of symptom onset.

**Figure 3:**
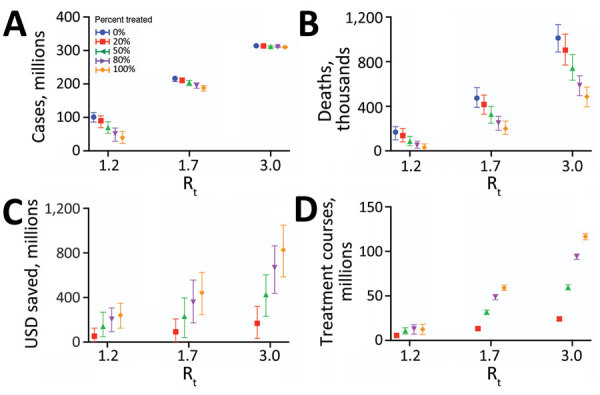
Projected health and economic impacts of a large-scale campaign using Paxlovid to treat COVID-19 over 300 days in the United States, across a range of transmission and treatment scenarios. Points and error bars correspond to means and 95% CI in number of infections in millions (A), number of deaths in millions (B), net monetary benefit in billions USD assuming a treatment course cost of US $530 and willingness to pay per year of life lost averted of US $100,000 (C), and number of courses of Paxlovid administered in millions (D). Each graph provides results for 3 R_t_ and 5 different treatment scenarios: 0% (blue), 20% (red), 50% (green), 80% (purple), or 100% (orange) of symptomatic cases started a 5-day course of Paxlovid within 3 days of symptom onset. Distributions are based on 100 stochastic simulations for each scenario. The results are scaled assuming a US population of 328.2 million (*21*). R_t_, effective reproduction number; USD, US dollars.

We estimated the number of cases, hospitalizations, and deaths, as well as healthcare costs, averted under a range of transmission scenarios, in which we varied both the between-individual transmission rate of the virus and the proportion of case-patients who received rapid treatment with Paxlovid (Table 3; Figures 2,3). Under a low-transmission scenario in which the R_t_ of the virus is 1.2, we estimated that treating 20% of symptomatic cases with Paxlovid would avert 10.54 million (95% CI 3.03–21.12 million) cases, 280,000 (95% CI 30,000–590,000) hospitalizations, and 33,850 (95% CI 1,690–71,150) deaths in the United States over a 300-day period (Appendix Table 4). Assuming a cost of US $530 per course of treatment (*22*) and willingness to pay per YLL averted of US $100,000, we estimated that the optimal strategy is always the highest achievable treatment rate. A 20% treatment rate would be expected to yield an NMB of US $56.95 billion (95% CI $2.62–$122.63 billion) averted. 

**Table 3:** Projected health and economic impacts of a large-scale SARS-CoV-2 Paxlovid campaign, United States

Outcome	R_t_	Treatment rate, %	Mean (95% CI)
Infections averted, millions	1.2	20	10.54 (3.03–21.12)
		50	25.65 (12.59–41.19)
	1.7	20	4.25 (0.00–8.30)
		50	10.65 (5.77–16.70)
	3	20	0.67 (−0.13 to 1.45)
		50	1.68 (0.79–2.77)
Hospitalizations averted, millions	1.2	20	0.28 (0.03–0.59)
		50	0.67 (0.33–1.25)
	1.7	20	0.48 (0.07–0.92)
		50	1.16 (0.49–1.85)
	3	20	0.85 (0.36–1.38)
		50	2.08 (1.12–2.83)
Deaths averted, thousands	1.2	20	33.85 (1.69–71.15)
		50	79.11 (35.78–146.51)
	1.7	20	59.43 (9.13–129.86)
		50	145.44 (45.60–221.34)
	3	20	109.67 (35.95–179.83)
		50	266.69 (156.71–362.77)
NMB, USD billions	1.2	20	$56.95 ($2.62–$122.63)
		50	$135.60 ($62.52–$261.32)
	1.7	20	$95.66 ($8.54–$196.23)
		50	$232.35 ($80.45–$379.51)
	3	20	$170.17 ($60.49–$286.14)
		50	$417.18 ($208.34–$580.13)
Courses of treatment used, millions	1.2	20	5.77 (4.38–7.15)
		50	12.13 (8.86–14.89)
	1.7	20	13.57 (12.42–15.12)
		50	32.85 (30.87–34.76)
	3	20	24.41 (22.34–26.56)
		50	60.21 (57.07–63.16)

*For each combination of treatment rate and reproduction number, the table provides the estimated mean and 95% CI of cases, hospitalizations, and deaths averted in the United States, NMB, and number of courses of treatment administered based on 100 pairs of stochastic simulations (treatment vs. no treatment simulations). NMB, net monetary benefit; R_t_, effective reproduction number; USD, US dollars.

To separate the direct (therapeutic) benefits of Paxlovid treatment from its indirect (transmission-reducing) effects, we conducted 2 additional analyses, 1 assuming the drug reduces severity but not infectivity and another assuming the opposite (Appendix Table 4). Assuming an R_t_ of 1.2, we estimated that direct therapeutic effects of treating 20% of symptomatic cases with Paxlovid would not affect the overall attack rate but would avert 140,000 (95% CI −130,000 to 400,000) hospitalizations and 16,470 (95% CI −19,470 to 48,110) deaths over a 300-day period, resulting in an NMB of US $25.35 (95% CI −$34.98 to $84.22) billion. The reduced infectivity of the treated cases would be expected to avert an additional 10.57 (95% CI 3.03–21.19) million infections, 160,000 (95% CI −130,000 to 530,000) hospitalizations, and 19,460 (95% CI −14,140 to 58,520) deaths, resulting in an NMB of US $31.17 (95% CI −$32.77 to $103.74) billion.

## Discussion

Our results show that the widespread administration of Paxlovid would not only improve outcomes in treated patients but also concomitantly reduce risks of onward transmission. In this population-level assessment of expanding rapid treatment of symptomatic COVID-19 infections with Paxlovid, we found that the direct (therapeutic) effects of treatment would substantially reduce both deaths and socioeconomic costs. Of note, the indirect (transmission-blocking) effects would be expected to reduce burden by just as much, as well as substantially reducing the overall attack rate (Appendix Table 4). We would expect mass treatment campaigns to have even greater health and economic effects in countries that have adopted zero-COVID strategies and thus have lower levels of population-level immunity than the United States (*23*).

Drugs like Paxlovid could profoundly reduce the severity of COVID-19 and enable a global transition to manageable coexistence with the virus. However, providing equitable and effective global access to SARS-CoV-2 antiviral drugs would require both ample supplies and broad-reaching test-and-treat programs. The pharmaceutical industry and global health agencies are working to produce enough Paxlovid to treat a large fraction of symptomatic cases (*8*). Online healthcare services (e.g., telemedicine) and community test-to-treat programs (*24*), such as those piloted in Pennsylvania and New Jersey (*25*), could be expanded nationally, and even globally, to accelerate and broaden access to antiviral drugs (*26*). For example, in 2020, China began an initiative to expand remote internet-based COVID-19 care (*27*). The country established 1,500 internet hospitals (either by extending existing hospitals or by opening new institutions) during 2019–2021 (*28*). The new services included follow-up consultations for common ailments (*29*) and served >239 million patients during December 2020–June 2021 (*30*). In addition, avoiding testing and treating infected individuals in person reduces the risk for SARS-CoV-2 transmission by patients to healthcare providers.

We highlight 3 limitations of our analyses that could be addressed as additional epidemiologic and clinical trial data become available. First, our fitted within-host model slightly overestimated viral levels for patients treated with placebo and underestimated those for patients receiving Paxlovid. The discrepancies might stem from limitations in the model structure or from unmodeled variation in viral kinetics and treatment efficacy across age or risk groups. In estimating model parameters, we considered only the mean in viral load of patients from 20 countries (*4*,*31*) (Figure 1). Incorporating such variability would enable us to analyze age-prioritized or risk-prioritized interventions and improve our estimates of the health and economic benefits of mass treatment. Second, we did not consider the emergence and spread of Paxlovid-resistant viruses, which could substantially undermine the utility of new drugs and exacerbate epidemics on a population level (*32*). Conversely, suppressed viral replication attributable to Paxlovid might limit viral evolution in treated patients. Depending on the immunological conditions of the individual person and population, reducing opportunities for viral growth and mutations could hinder the emergence of new variants (*33*). Third, we did not incorporate several economic, social, and logistical factors that might affect the expansion of Paxlovid treatment, including commercial impediments faced by the pharmaceutical companies that manufacture the drug (*34*); the costs of administering tests before treatment; and low levels of uptake stemming from misinformation, limited healthcare access, or pandemic fatigue. For example, in the 2009 H1N1 pandemic, only 40% of case-patients sought medical care within 3 days after symptom onset (*35*). 

In conclusion, fast-acting antiviral drugs like Paxlovid can serve as invaluable tools to mitigate COVID-19 epidemics. By increasing supplies and improving infrastructure to enable rapid and equitable distribution, such drugs could substantially mitigate the health and societal burdens of COVID-19.

Appendix Additional information about public health impact of Paxlovid as treatment for COVID-19, United States
