# Supplementary material for: Public Health Impact of Paxlovid as Treatment for COVID-19, United States
Source: Emerg Infect Dis. 2024 Feb;30(2):262–9. doi: 10.3201/eid3002.230835 (PMC10826746; doi:10.3201/eid3002.230835)
Supplement: Appendix — Additional information about public health impact of Paxlovid as treatment for COVID-19, United States [file 23-0835-Techapp-s1.pdf]

Article DOI: https://doi.org/10.3201/eid3002.230835

EID cannot ensure accessibility for supplementary materials supplied by authors. Readers who have difficulty accessing supplementary content should contact the authors for assistance.

Public Health Impact of Paxlovid as Treatment for COVID-19, United States

Appendix

Within-Host Model of SARS-CoV-2 Replication Dynamics

The deterministic model given by

$$\frac{dU_i}{dt} = -bU_iV_i$$

$$\frac{dI_i}{dt} = bU_iV_i - \delta I_i$$

$$\frac{dV_i}{dt} = (1 - \epsilon)pI_i - cV_i$$

tracks the number of target cells at risk of infection (U_i), infected cells (I_i), and free viral particles (V_i) (*1*) (Appendix Figure 1). The rate at which free viral particles infect target cells is governed by the number of susceptible target cells, the number of free viral particles, and a fixed rate b. Viruses replicate at a rate p in infected cells; infected cells die at rate δ and free viral particles die at rate c. The model assumes that Paxlovid inhibits the replication of viruses within infected cells, with efficacy ϵ.

Estimating the within-host model parameters

We fix the initial number of viruses (V_0) at 1/30 copy/mL (corresponding to a single viral particle per 30 mL of nasal wash in the upper respiratory tract (*2*)) and the initial number of target cells (U_0) at 10^7 (*1*). For the Delta variant, the estimated average time from infection to symptom onset is 5 days (*3*); in recent clinical trials, the estimated average time from symptom

onset to initiation of treatment is 3 days (*4*). Based on these estimations, we assume that treatment is initiated 8 days after infection for treated patients.

To estimate the five model parameters governing the viral load dynamics (i.e., the infection rate of susceptible cells [b], the rate at which infected cells die [δ], the rate at which active viruses were cleared [c], the virus production rate [p] and antiviral efficacy [ϵ]), we fit the within-host model to the mean SARS-Cov-2 RNA titer (log10 copies/mL) at five time points (1, 3, 5, 10 and 14 days post initiation of treatment) measured across 1126 infected adults treated with a placebo during a clinical trial in late 2021 (*4*) and the mean SARS-Cov-2 RNA titer (log10 copies/mL) at five time points (1, 3, 5, 10 and 14 days post initiation of treatment) measured across 1120 infected adults who received Paxlovid in the same clinical trial (*5*). We set the initial viral load upon infection, V_0, to correspond to one infectious virus particle in the upper respiratory tract (*2*). We assume that the average viral load at the initiation of treatment is 10^6 log10 copies/mL (*4*). We use the Stochastic Approximation Expectation-Maximization (SAEM) algorithm to estimate the five parameters (MONOLIX 2021R1) (*6*,*7*) and confirm the convergence of estimates via trace plots.

Modeling the daily infectiousness of treated and untreated cases

Our between-host SARS-CoV-2 transmission model assumes that the infectiousness of an infected individual depends on the number of days elapsed since they became infected (t), whether or not they receive Paxlovid, and, if so, how quickly treatment is initiated. We use ι to indicate treatment initiation time in days after symptom onset and $\iota = \infty$ to denote that a case remains untreated. Specifically, we assume that an individual's infectiousness *is proportional to*:

$$\beta_\iota = \log(V_\iota(t))$$

Where $V_\iota(t)$ represents the individual's viral load t days after infection. We use the fitted within-host model above to generate the $V_\iota(t)$, depending on whether and when the infected individual receives Paxlovid and assuming that infectiousness drops to zero when the viral load drops below the detection threshold of 100 (*8*).

Between-host model of SARS-CoV-2 transmission and treatment with Paxlovid-like drug

Our between-host agent-based model assumes that an infected individual's daily infectiousness toward one of their contacts depends on: (i) the time elapsed since infection, (ii) whether and when Paxlovid treatment is initiated, and (iii) whether the case and contact live in the same household. We use the infectiousness equation above (β_ι) to account for the first two variables, and then solve for household and non-household scaling constants that yield target secondary infection rates. Specifically, we first calibrate the daily within-household transmission rates for untreated cases to match reported estimates for household secondary attack rates (which is constant across all scenarios). We then calibrate the daily non-household transmission rates for untreated cases so that the model produces a specified overall initial reproduction number (which depends on the scenario analyzed).

To calibrate the within-household transmission rate scaling constant (ξ_h), we assume that a household secondary attack rate of 35% (*9*) and solve for the ξ_h that satisfies $\xi_h \sum_{t=0}^{50} \beta_\infty(t) = 0.35$. To calibrate the non-household transmission rate scaling constant (ξ_{nh}), we set the target initial effective reproduction number ($\bar{R}_e$) and then apply an interior point algorithm to find the value of ξ_{nh} that minimizes the mean square error between $\bar{R}_e$ and the average initial R_e across 100 simulated epidemics. For each simulation, we estimate R_e by calculating the average number of secondary infections across a random sample consisting of 1% of individuals infected during the first 100 days of the simulation.

At the start of a simulation, we set the proportions of the population with infection-acquired and vaccine-acquired immunity to values estimated from data provided by the U.S. Centers for Disease Control and Prevention (Appendix Table 1). To estimate the number of previously vaccinated individuals and the date of their most recent dose, we simulated vaccination rates based on reported uptake in the U.S. from 2020 to 2022 (*10*). For each previously vaccinated individual, we randomly selected the date of their first dose (t_1) based on the reported age-specific vaccine administration rates, starting on October 29, 2021 (*11*) for children between 5 and 11 years old, May 10, 2021 for children between 12 and 15 years old (*12*), and December 13, 2020 for all others. We then randomly determined whether and when an individual receives their second primary dose and first booster based on CDC-recommended

waiting periods and reported rates of uptake. Specifically, we assumed second doses are administered beginning 3 weeks after the first dose, and the window for boosters depends on the timing of the booster dose, with a minimum gap of 8 months for individuals receiving their booster dose before September 23, 2021 (*13*), 6 months between September 24, 2021 and January 3, 2022 (*14*), and 5 months after January 4, 2022 (*15*). We initialized immunity in our simulations using the dates of the last dose received for each vaccinated individual (Appendix Table 1) (*16*).

For the previously infected individuals, we estimated their times of recovery. Specifically, we collected the daily population proportion of confirmed cases in the USA from 2021 to 2022 from Our World In Data (*10*). For each individual infected previously at the start of the simulation, we estimated the date of the previous infection (t_{infect}) by taking draws from the distribution of the daily population proportion of cases between January 29, 2021 to January 29, 2022. We considered the time of recovery as (t_{infect} + 9), where 9 days is the average time lag between infection and recovery (*17*).

At the start of each simulation, we also assume that 1% of the unvaccinated susceptible and recovered populations are newly infected (exposed), which corresponds to ≈0.6% of the total population. We assumed age-stratified estimates for Paxlovid's efficacy at preventing hospitalizations (*18*) (Appendix Table 1) and incorporated uncertainty by sampling the Paxlovid efficacy parameters for each simulation from triangular distributions with mean, lower bound, and upper bound equal to the estimated mean, 95% CI lower bound, and 95% CI upper bound, respectively. To estimate therapeutic benefits of the drug via pairs of simulations, we enforced the same sequence of random numbers in each simulation.

Individual-based network construction

The individual-based SARS-CoV-2 infection dynamic model assumes that the virus spreads through a fixed contact network consisting of 9961 individuals and 124,878 contacts between those individuals. We populated our network by first constructing 5000 households. The size and age composition of each household is based on a randomly sampled household from among the 129,697 households included in 2017 National Household Travel Survey (*19*). We assumed that households are fully connected (i.e., all nodes in the same household are linked by

edges). We constructed random links between individuals in different households based on reported age-specific contact rates in the U.S., stratified into age bins of 5–17, 18–49, 50–64, and over 65 years (*20*). Specifically, to determine the number of contacts a node in age group a_i has with nodes in age group a_j, we draw random deviates from Poisson distributions centered at the mean number of contacts between a_i and a_j. The resulting network includes 5000 households, 2019 nodes (people), and degrees (numbers of edges per node) that roughly follow a gamma distribution with shape and scale parameters of 3.69 and 3.41, respectively. We directly scaled our results to the 2019 U.S. population of 328 million (*21*).

Estimating the Years of Life Lost (YLL) Averted and Monetary Costs

For each set of stochastic simulations, we estimated the years of life loss (YLL) averted for each antiviral strategy τ as follows:

1. Calculate the difference in incidence by age group as $\Delta_{a,\tau} = D_{a,0} - D_{a,\tau}$, where $D_{a,0}$ and $D_{a,\tau}$ are total deaths in age group *a* produced by the no treatment and strategy τ simulations, respectively.

2. Estimate the YLL prevented by the strategy τ as $B_\tau = \sum_a (\lambda_a - a)\Delta_{a,\tau}$ where λ_a denotes the future-discounted life expectancy for individuals of age *a*.

Similarly, we determined the incremental monetary costs for each strategy τ as given by

$$C_\tau = (T_\tau - T_0)c_T + \sum_a c_{H,a}(H_{\tau,a} - H_{0,a})$$

where T_0 and T_τ are the total number of treatment courses administered in the no treatment and strategy τ simulations, respectively, c_T is the price of administering one course of antivirals, $H_{\tau,a}$ and $H_{0,a}$ are the total number of hospitalizations in age group *a* in each simulation, and $c_{H,a}$ is the median COVID-19 hospitalization cost for age group a. The cost parameter values are given in Appendix Table 3.

For a given willingness to pay for a YLL averted (θ), we calculated the net monetary benefit (NMB) of a strategy as

$$NMB_\tau = \theta \cdot B_\tau - C_\tau.$$

We determined the optimal strategy across a range of scenarios, each defined by the effective reproduction number (R_e), willingness to pay, and cost of a vaccine.

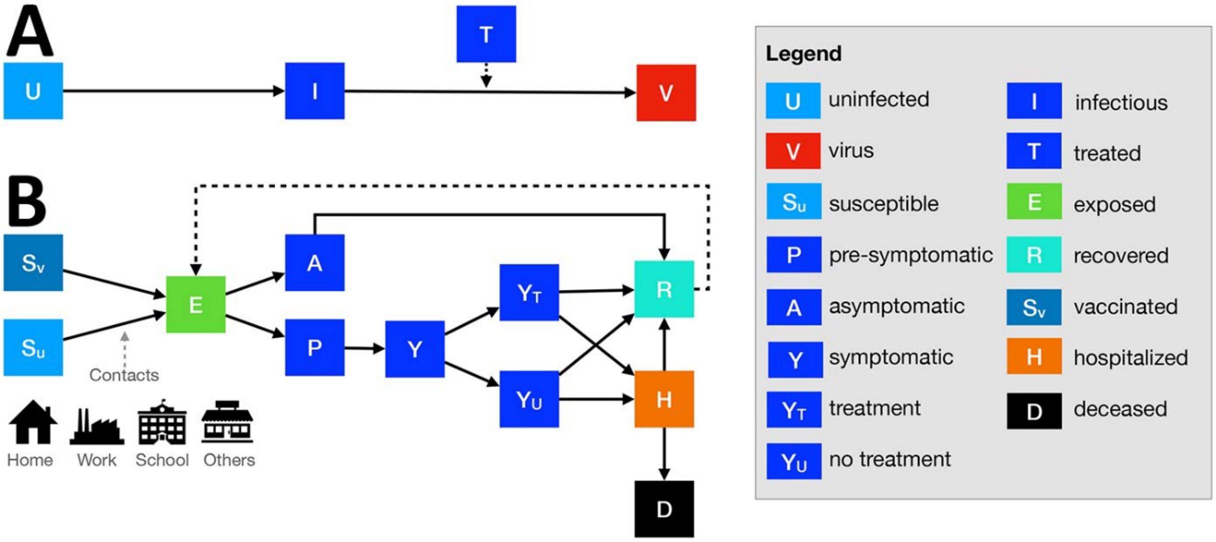

Appendix Figure 1. Diagrams of the within-host and between-host models. (A) To estimate changes in infectivity following treatment with Paxlovid (T), we used a model that tracks the changing number of uninfected cells (U), infectious cells (I) and free viral particles (V) in an infected case, with and without treatment. (B) We projected population-level impacts of Paxlovid treatment using a stochastic individual-based model of SARS-CoV-2 transmission that considers age-specific risks and contact patterns in households, schools, workplaces, and other venues. Upon infection, susceptible (S_u) and vaccinated (S_v) individuals progress to exposed (E), asymptomatic infectious (A) or presymptomatic (P) and then to either symptomatic infectious (Y) with (Y_T) or without (Y_U) Paxlovid treatment. A fraction of symptomatic cases with or without treatment will be recovered (R) or hospitalized (H), and a subset of those will die (D). All asymptomatic cases eventually progress to a recovered class (R), where they remain protected from future infection. As immunity wanes, recovered individuals return to exposed (E) by reinfection.

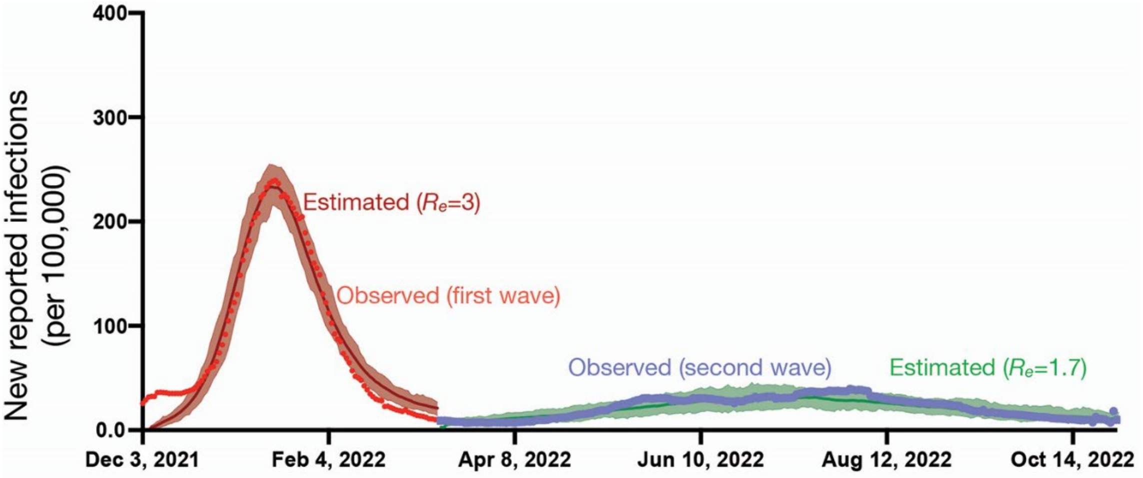

Appendix Figure 2. Comparison of observed (*22*) to estimated SARS-CoV-2 incidence during two Omicron waves in the U.S.. Model estimates assume an initial effective reproduction number of 3.0 for the first large Omicron wave in the U.S. (December 3, 2021 to March 12, 2022) and a reproduction number of 1.7 for the second smaller wave (March 13, 2021 to October 31, 2022), and assume a case reporting rate of 25% (*23*). Points indicate reported incidence data; lines and shading correspond to the mean and 95% confidence interval across 100 stochastic simulations for each wave.

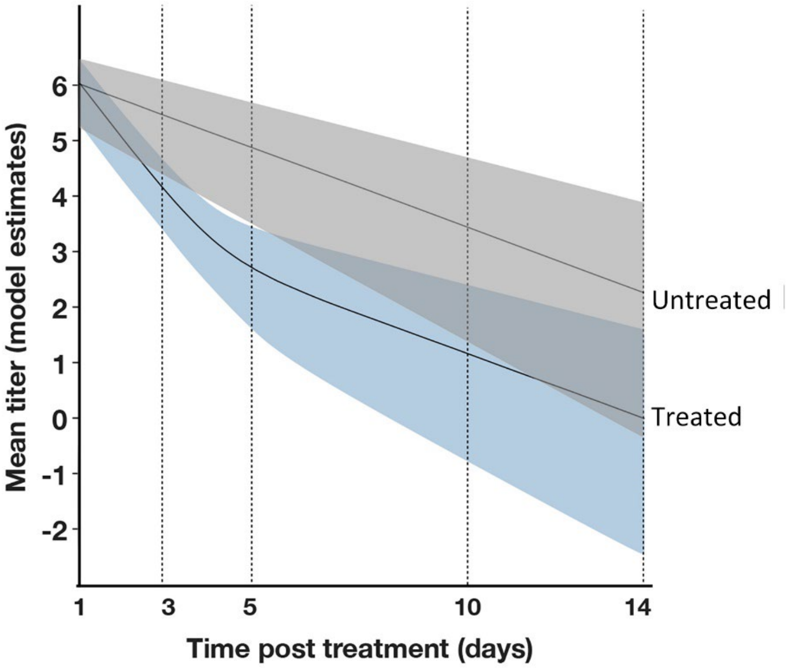

Appendix Figure 3. Comparison between the estimated mean viral loads for treated versus untreated cases. Lines and shading indicate the means and 95% confidence intervals of SARS-CoV-2 viral load (RNA log10 copies/mL) as estimated by the fitted within-host model. Day one corresponds to the initiation of treatment.

Appendix Table 1. Parameter values.

Parameters	Values	Source
N: number of individuals in the population	9961 individuals in 5000 households	
Initial percent vaccinated as of January 29, 2022 for [0–4y, 5–17y, 18–49y, 50–64y, >65y]	At least one dose: [0, 42.4%, 77.2%, 89.6%, 97.4%]<br>One dose only: [0%, 4.50%, 2.30%, 1.70%, and 1.20%]<br>Primary series only (two doses): [0%, 37.90%, 29.60%, 25.80%, and 21.30%]<br>Primary and first booster: [0%, 0%, 45.30%, 62.10%, and 74.90%]	(*16*)
Initial infections (in exposed state)	1% of the susceptible population	Assumed
Initial proportion of recovered	[39.34%, 60.81%, 50.61%, 48.38%, and 35.88%] for [0–4y, 5–17y, 18–49y, 50–64y, >65y]	(i) 77M, 129M, and 220M reported cases in the U.S. on 29 January 2021, 30 September 2021, and 29 January 2022, respectively (*10*). (ii) U.S. CDC estimates [35.49%, 54.86%, 45.66%, 43.65%, 32.36%] for [0–4y, 5–17y, 18–49y, 50–64y, >65y] from February 2020 to September 2021 (*24*). (iii) Thus we set the age-specific proportions of the U.S. population infected between January 29, 2021 and January 29, 2022 to (220–77)/129.*[35.49%, 54.86%, 45.66%, 43.65%, 32.36%].
ψ: symptomatic proportion (%)	75	(*25*)
ρ: treatment proportion (%)	Depends on scenario	Assumed
ξ_{nh}: baseline transmission rate	ξ_{nh} is 0.0004, 0.0005, 0.0006, 0.0008, and 0.0013 for R_e of 1.2, 1.5, 1.7, 2, and 3, respectively.	Calibrated to R_e.
σ: transition rate out of exposed state (d^{-1})	1/3	(*3*)
γ_A: asymptomatic recovery rate (d^{-1})	1/9	(*17*)
γ_Y: symptomatic recovery rate (d^{-1})	1/4	(*26*,*27*)
ω: transition rate from the pre-symptomatic to the symptomatic stage (d^{-1})	1/2	(*3*)
h_a: age-specific proportion of symptomatic cases that are hospitalized	[0%, 0.025%, 2.672%, 9.334%, 15.465%] for [0–4y, 5–17y, 18–49y, 50–64y, >65y]	(*28*)
φ_a: age-specific efficacy of Paxlovid in reducing the hospitalization rate	[0.59 (95% CI: 0.48, 0.71), 0.59 (95% CI: 0.48, 0.71), 0.59 (95% CI: 0.48, 0.71), 0.40 (95% CI: 0.34, 0.48), 0.53 (95% CI: 0.48, 0.58)] for [0–4y, 5–17y, 18–49y, 50–64y, >65y]	(*18*)<br>The study provides estimates for adults over age 18y. We assumed that efficacy for children under 18 is the same as that for adults aged 18–49y.
η: transition rate from treatment to hospitalized (d^{-1})	$1/(5.9–1/\gamma_T)$	5.9 d on average from symptomatic to hospitalized (*29*)
γ_T: transition rate from symptomatic to treatment (d^{-1})	1/3	an average of 3 d between COVID-19 symptom onset and the initiation of Paxlovid treatment (*4*)
μ_a: age-specific mortality rate for hospitalized cases	[0.48%, 0.48%, 5.68%, 10.82%, 16.15%] for [0–4y, 5–17y, 18–49y, 50–64y, >65y]	(*30*)
γ_d: transition rate from hospitalized to deceased for cases that succumb (d^{-1})	0.128	(*31*)
γ_h: transition rate from hospitalized to recovered for cases discharged alive (d^{-1})	0.091	(*31*)
λ_a: life expectancy (years) for age group *a*, adjusted assuming a 3% yearly discount rate	[30.3, 29.3, 25.8, 18.7, 12.9] for [0–4y, 5–17y, 18–49y, 50–64y, >65y]	(*32*)

Appendix Table 2. Parameters governing waning of immunity following vaccination and infection with respect to the SARS-CoV-2 Omicron variant. Vaccine-related parameter values are based on estimates for the BNT162b2 (Pfizer) vaccine.

	Immunity following vaccination		
Time since most recent vaccination	Reduction in susceptibility to infection (ω_B)	Reduction in likelihood of developing symptoms following infection (ψ_B)	Reduction in risk of mortality following infection (θ_B)
1 week	64%*	66.9% (73)	91.9% *
2 to 4 weeks	64%*	67.2% (73)	91.9%*
5 to 9 weeks	42.8%*	55.0% (73)	91.9%*
> = 10 weeks	22%*	45.7% (73)	91.9%*
Time since recovery	Immunity following infection		
	Reduction in susceptibility to infection (ω_N)	Reduction in likelihood of developing symptoms following infection (ψ_N)	Reduction in risk of mortality following infection (θ_N)
1 week	83.1%+	92.1%++	98.1%++
2 to 4 weeks	83.1%+	92.1%++	98.1%++
5 to 9 weeks	73.1%+	89.2%++	98.1%++
> = 10 weeks	63.3%+	87.0%++	98.1%++

* Since direct estimates of ω_B and θ_B for vaccine booster doses against the Omicron variant are not available, we extrapolated from Ref (*33*). which estimates ω_B for boosters against Omicron and other studies that simultaneously estimate vaccine efficacy against infection, symptomatic infection, and mortality for the Pfizer-BioNTech BNT162b2 vaccine earlier in the pandemic. In Ref (*34*), vaccine efficacy against infection is estimated to be 64% when efficacy against symptomatic disease reaches 67% (21 d after vaccination). In Ref (*35*), vaccine efficacy against infection is estimated to be 42.8% when efficacy against symptomatic disease reaches 52.4% (14 d after the initial vaccine dose). In Ref (*34*), vaccine efficacy against infection is estimated to be 22% when efficacy against symptomatic disease reaches 44% (14 d after the initial vaccine dose). In Ref (*36*), vaccine efficacy against mortality is estimated to be 91.9% when efficacy against symptomatic disease reaches 66.3% (20 d after the second vaccine dose).

** Since direct estimates for ψ_N against the Omicron variant are not available, we extrapolated from Ref (*37*). which estimates ω_N against Omicron and Ref (*38*), which reports that vaccine efficacy against symptomatic disease is 97% when efficacy against infection reaches 79% (14 d after the second vaccine dose).

+ Using an estimated adjusted hazard ratio of SARS-CoV-2 infection following natural infection versus BNT162b2 vaccination of 0.47 (95% CI: 0.45–0.48), we scaled ω_N based on ω_B (*39*).

++ Using an estimated adjusted hazard ratio of severe SARS-CoV-2 infection following natural infection versus BNT162b2 vaccination of 0.24 (95% CI: 0.08–0.72), we scaled ψ_N based on ψ_B (*39*) and θ_N based on θ_B.

Appendix Table 3. Cost parameters.

Parameter	Value (USD)
Cost of administering a treatment course (c_T) by taking Paxlovid	$530 per course (*40*)
Median COVID-19 hospitalization cost by age group ($c_{H,a}$)	[$21,847, $21,847, $19,681, $23,157, $18,806] for [0–4y, 5–17y, 18–49y, 50–64y, >65y] (*41*)

Appendix Table 4. Projected cases, hospitalizations and deaths averted in the U.S., net monetary benefit (billion USD), and treatment courses administered in a large-scale SARS-CoV-2 antiviral campaign, across four treatment rates and five transmission scenarios (effective reproduction numbers from 1.2 to 5). Assuming a treatment course cost of U.S.$530 and willingness to pay (WTP) per year of life lost (*YLL*) averted of U.S.$100,000, for each reproduction number and treatment rate, we estimated the mean and 95% confidence intervals based on 100 pairs of stochastic simulations (treatment vs. no treatment simulations). To separate the direct therapeutic benefits of the drug from the indirect transmission-blocking impacts of treatment, we analyzed an alternative model in which the drug improves patient outcomes but does not impact infectivity.

			Mean (95% CI)		
Outcome	R_e	Treatment rate (% symptomatic cases)	Alternative model: indirect (transmission reducing) effect only	Alternative model: direct (therapeutic) effects only	Base model
No. of cases averted (million)	1.2	20%	10.57 (3.03, 21.19)	−0.22 (−3.82, 1.91)	10.54 (3.03, 21.12)
		50%	25.99 (12.88, 45.11)	−0.32 (−4.84, 2.37)	25.65 (12.59, 41.19)
		80%	43.13 (25.30, 80.72)	−0.61 (−5.34, 2.90)	42.58 (25.24, 67.45)
		100%	54.21 (36.77, 81.94)	−0.84 (−6.19, 3.26)	53.74 (36.67, 81.88)
	1.5	20%	4.92 (0.07, 9.32)	−0.28 (−2.14, 0.99)	4.85 (−0.03, 9.29)
		50%	13.76 (7.55, 19.54)	−0.44 (−3.69, 1.98)	13.55 (7.51, 19.21)
		80%	23.71 (16.38, 30.21)	−0.76 (−4.68, 2.54)	23.43 (16.21, 29.92)
		100%	31.33 (23.43, 41.35)	−0.84 (−4.61, 2.17)	30.84 (23.39, 40.69)
	1.7	20%	4.26 (0.07, 8.37)	−0.13 (−2.14, 1.45)	4.25 (0.00, 8.30)
		50%	10.87 (6.16, 16.70)	−0.33 (−2.64, 1.91)	10.65 (5.77, 16.70)
		80%	18.39 (10.54, 24.41)	−0.58 (−2.73, 1.52)	18.15 (10.51, 24.09)
		100%	23.87 (16.47, 30.11)	−0.57 (−2.73, 1.32)	23.50 (15.85, 29.98)
	2	20%	2.93 (−0.23, 6.26)	−0.10 (−1.65, 1.55)	2.86 (−0.26, 6.16)
		50%	7.14 (3.03, 11.66)	−0.39 (−1.94, 1.05)	6.96 (3.00, 11.73)
		80%	12.01 (7.15, 18.62)	−0.71 (−2.77, 0.82)	11.63 (6.89, 18.48)
		100%	15.55 (11.27, 21.38)	−0.86 (−3.00, 1.19)	15.19 (10.81, 20.99)
	3	20%	0.71 (−0.07, 1.45)	−0.05 (−0.49, 0.26)	0.67 (−0.13, 1.45)

Outcome	R_e	Treatment rate (% symptomatic cases)	Mean (95% CI) Alternative model: indirect (transmission reducing) effect only	Alternative model: direct (therapeutic) effects only	Base model
		50%	1.74 (0.79, 2.83)	−0.13 (−0.76, 0.40)	1.68 (0.79, 2.77)
		80%	2.92 (1.94, 4.18)	−0.21 (−0.82, 0.43)	2.80 (1.78, 4.02)
		100%	3.77 (2.67, 5.07)	−0.24 (−0.79, 0.46)	3.67 (2.67, 5.01)
	5	20%	0.03 (−0.10, 0.13)	0.00 (−0.07, 0.03)	0.03 (−0.07, 0.13)
		50%	0.08 (−0.07, 0.20)	0.00 (−0.10, 0.10)	0.07 (−0.07, 0.20)
		80%	0.11 (−0.07, 0.33)	−0.01 (−0.10, 0.07)	0.11 (−0.07, 0.30)
		100%	0.14 (−0.07, 0.33)	−0.02 (−0.13, 0.10)	0.12 (−0.07, 0.33)
Deaths averted (thousand)	1.2	20%	19.46 (−14.14, 58.52)	16.47 (−19.47, 48.11)	33.85 (1.69, 71.15)
		50%	48.14 (−8.82, 117.86)	43.36 (1.58, 91.31)	79.11 (35.78, 146.51)
		80%	76.95 (18.23, 148.21)	65.25 (17.88, 121.49)	113.96 (53.68, 166.28)
		100%	95.19 (20.40, 165.92)	81.27 (17.71, 143.13)	133.31 (60.98, 185.92)
	1.5	20%	13.07 (−49.99, 78.46)	36.87 (−13.91, 86.89)	50.23 (−1.93, 114.14)
		50%	35.75 (−48.24, 121.17)	94.77 (5.56, 164.45)	123.79 (39.24, 201.48)
		80%	61.77 (−71.70, 162.12)	150.07 (66.01, 238.88)	188.19 (89.04, 277.98)
		100%	82.50 (−48.31, 178.02)	186.98 (83.38, 276.20)	231.40 (124.93, 335.46)
	1.7	20%	11.93 (−44.68, 78.58)	48.49 (7.54, 104.72)	59.43 (9.13, 129.86)
		50%	35.10 (−62.19, 134.72)	120.77 (28.96, 197.79)	145.44 (45.60, 221.34)
		80%	52.69 (−43.96, 174.65)	190.28 (94.44, 297.07)	221.79 (115.52, 315.25)
		100%	70.01 (−40.05, 211.69)	238.48 (136.21, 349.11)	272.44 (180.06, 392.08)
	2	20%	9.02 (−78.52, 74.64)	61.73 (5.45, 117.52)	70.75 (4.05, 144.58)
		50%	21.92 (−76.91, 129.39)	153.74 (64.08, 240.41)	174.36 (83.69, 271.33)
		80%	44.70 (−75.05, 166.76)	244.56 (108.42, 341.85)	275.73 (158.47, 377.52)
		100%	57.57 (−94.86, 173.98)	303.84 (158.23, 408.09)	338.93 (209.58, 456.58)
	3	20%	7.76 (−109.69, 76.28)	103.03 (41.24, 174.82)	109.67 (35.95, 179.83)
		50%	16.97 (−91.08, 123.79)	257.11 (148.62, 362.85)	266.69 (156.71, 362.77)
		80%	31.25 (−113.81, 158.07)	404.90 (266.90, 515.39)	425.24 (277.20, 552.68)
		100%	41.88 (−109.69, 182.87)	507.09 (359.15, 646.19)	525.51 (384.94, 663.72)
	5	20%	0.90 (−120.85, 116.60)	154.32 (66.30, 259.38)	161.81 (56.01, 261.83)
		50%	−0.16 (−175.10, 154.01)	387.76 (231.08, 547.49)	399.03 (246.21, 562.19)
		80%	2.05 (−263.34, 205.53)	620.23 (444.13, 801.75)	632.56 (447.52, 812.49)
		100%	−6.88 (−310.29, 245.90)	768.62 (569.70, 997.59)	782.03 (586.95, 1002.60)
NMB (billion USD)	1.2	20%	31.17 (−32.77, 103.74)	25.35 (−35.19, 84.22)	56.95 (2.62, 122.63)
		50%	80.36 (−24.60, 205.73)	68.03 (−6.92, 151.96)	135.60 (62.52, 261.32)
		80%	130.70 (26.22, 254.08)	102.12 (13.07, 189.75)	197.15 (97.62, 293.82)
		100%	163.44 (22.57, 288.36)	126.96 (16.21, 228.25)	232.26 (107.17, 332.85)
	1.5	20%	15.55 (−90.17, 134.55)	58.18 (−19.35, 127.21)	81.07 (−10.22, 194.06)
		50%	45.58 (−99.51, 193.46)	149.22 (−1.33, 276.58)	201.38 (50.16, 327.12)
		80%	83.46 (−140.08, 282.27)	238.03 (85.13, 389.40)	307.42 (125.44, 449.21)
		100%	115.04 (−99.49, 299.60)	295.70 (111.03, 447.24)	378.72 (198.82, 560.47)
	1.7	20%	12.67 (−82.30, 133.89)	76.14 (5.94, 157.33)	95.66 (8.54, 196.23)
		50%	39.00 (−130.16, 170.20)	190.12 (32.17, 332.79)	232.35 (80.45, 379.51)
		80%	60.66 (−112.13, 257.44)	300.90 (109.99, 451.75)	356.40 (176.38, 499.36)
		100%	86.49 (−102.48, 328.03)	377.20 (196.17, 547.91)	439.82 (266.32, 610.84)
	2	20%	4.57 (−122.98, 126.50)	96.74 (3.28, 194.97)	111.39 (4.57, 246.24)
		50%	14.18 (−169.44, 191.31)	240.68 (78.62, 396.23)	276.52 (97.96, 459.36)
		80%	41.73 (−184.27, 232.06)	385.48 (139.63, 559.60)	439.08 (208.27, 618.77)
		100%	54.86 (−194.98, 282.01)	478.73 (205.19, 687.67)	539.73 (309.83, 764.58)
	3	20%	−2.66 (−172.88, 125.58)	159.56 (62.97, 284.89)	170.17 (60.49, 286.14)
		50%	−4.31 (−179.49, 193.58)	401.32 (219.88, 578.78)	417.18 (208.34, 580.13)
		80%	1.64 (−239.00, 234.46)	633.73 (384.92, 820.60)	665.92 (414.82, 878.32)
		100%	5.43 (−272.37, 270.19)	789.52 (541.89, 982.78)	821.12 (595.37, 1059.33)
	5	20%	−24.98 (−212.90, 142.87)	234.19 (101.47, 417.79)	247.08 (92.89, 409.43)
		50%	−53.96 (−348.55, 188.46)	596.98 (351.54, 838.32)	616.81 (366.77, 863.68)
		80%	−77.94 (−500.47, 261.01)	960.35 (646.44, 1267.53)	982.74 (685.66, 1298.68)
		100%	−112.37 (−612.92, 327.29)	1189.80 (860.17, 1567.85)	1214.09 (885.29, 1605.70)
Treatment courses used (million)	1.2	20%	5.77 (4.38, 7.08)	6.45 (4.78, 7.68)	5.77 (4.38, 7.15)
		50%	12.08 (7.31, 14.89)	16.24 (12.55, 18.42)	12.13 (8.86, 14.89)
		80%	14.90 (5.93, 20.92)	26.07 (21.35, 29.42)	15.04 (6.79, 21.02)
		100%	15.15 (4.94, 21.78)	32.68 (26.66, 36.80)	15.30 (5.93, 21.91)
	1.5	20%	11.64 (10.31, 12.88)	11.92 (10.84, 12.88)	11.64 (10.35, 12.92)
		50%	27.54 (25.60, 29.39)	29.74 (27.51, 31.73)	27.57 (25.57, 29.36)
		80%	41.50 (38.68, 44.88)	47.60 (44.88, 50.28)	41.58 (38.78, 45.04)
		100%	49.52 (45.17, 53.01)	59.62 (56.41, 63.26)	49.69 (45.34, 52.98)
	1.7	20%	13.58 (12.42, 15.09)	13.88 (12.62, 15.42)	13.57 (12.42, 15.12)
		50%	32.81 (31.04, 34.73)	34.60 (32.55, 36.70)	32.85 (30.87, 34.76)

Outcome	R_e	Treatment rate (% symptomatic cases)	Mean (95% CI): Alternative model: indirect (transmission reducing) effect only	Alternative model: direct (therapeutic) effects only	Base model
		80%	50.70 (47.61, 53.54)	55.38 (52.09, 58.38)	50.74 (47.61, 53.90)
		100%	61.49 (58.02, 63.95)	69.26 (65.30, 73.05)	61.61 (58.22, 64.28)
	2	20%	16.32 (14.73, 17.96)	16.54 (14.83, 18.09)	16.32 (14.73, 18.02)
		50%	40.03 (37.96, 42.40)	41.40 (39.11, 44.35)	40.07 (37.86, 42.47)
		80%	62.78 (58.68, 66.36)	66.37 (62.57, 70.02)	62.89 (58.58, 66.19)
		100%	77.32 (72.98, 81.94)	83.17 (78.45, 87.68)	77.43 (72.98, 81.81)
	3	20%	24.42 (22.31, 26.62)	24.58 (22.50, 26.72)	24.41 (22.34, 26.56)
		50%	60.16 (57.00, 63.13)	61.43 (58.45, 64.41)	60.21 (57.07, 63.16)
		80%	94.96 (91.56, 98.71)	98.13 (94.92, 101.78)	95.06 (91.70, 98.68)
		100%	118.06 (114.50, 121.78)	122.84 (119.41, 126.75)	118.21 (114.89, 122.17)
	5	20%	38.42 (35.98, 41.09)	38.57 (36.08, 41.15)	38.43 (35.98, 41.19)
		50%	95.25 (91.20, 99.27)	96.23 (92.29, 100.16)	95.34 (91.50, 99.34)
		80%	151.64 (146.42, 156.47)	154.18 (150.34, 159.50)	151.84 (146.62, 156.83)
		100%	189.18 (184.81, 194.49)	193.11 (188.47, 198.65)	189.44 (185.27, 195.02)
Hospitalizations reduced (million)	1.2	20%	0.16 (−0.13, 0.53)	0.14 (−0.13, 0.40)	0.28 (0.03, 0.59)
		50%	0.41 (−0.07, 0.99)	0.36 (0.00, 0.76)	0.67 (0.33, 1.25)
		80%	0.65 (0.16, 1.22)	0.55 (0.10, 0.96)	0.96 (0.49, 1.42)
		100%	0.80 (0.16, 1.35)	0.68 (0.20, 1.15)	1.13 (0.56, 1.58)
	1.5	20%	0.10 (−0.40, 0.66)	0.30 (−0.03, 0.63)	0.41 (−0.03, 0.92)
		50%	0.28 (−0.40, 0.96)	0.77 (0.07, 1.38)	1.01 (0.30, 1.58)
		80%	0.48 (−0.43, 1.38)	1.23 (0.49, 1.91)	1.53 (0.66, 2.14)
		100%	0.65 (−0.36, 1.52)	1.53 (0.66, 2.24)	1.88 (0.99, 2.67)
	1.7	20%	0.09 (−0.33, 0.66)	0.39 (0.07, 0.79)	0.48 (0.07, 0.92)
		50%	0.26 (−0.49, 0.86)	0.97 (0.26, 1.68)	1.16 (0.49, 1.85)
		80%	0.40 (−0.43, 1.32)	1.54 (0.69, 2.27)	1.78 (0.89, 2.44)
		100%	0.55 (−0.36, 1.61)	1.93 (1.15, 2.73)	2.19 (1.38, 2.93)
	2	20%	0.06 (−0.46, 0.59)	0.49 (0.03, 0.92)	0.55 (0.10, 1.19)
		50%	0.16 (−0.72, 1.02)	1.22 (0.46, 1.94)	1.38 (0.59, 2.24)
		80%	0.34 (−0.76, 1.19)	1.95 (0.86, 2.80)	2.19 (1.12, 3.00)
		100%	0.44 (−0.69, 1.42)	2.43 (1.19, 3.43)	2.69 (1.65, 3.72)
	3	20%	0.05 (−0.69, 0.59)	0.80 (0.40, 1.32)	0.85 (0.36, 1.38)
		50%	0.13 (−0.63, 1.12)	2.01 (1.15, 2.83)	2.08 (1.12, 2.83)
		80%	0.24 (−0.82, 1.35)	3.17 (2.08, 4.05)	3.30 (2.14, 4.22)
		100%	0.31 (−0.89, 1.48)	3.95 (2.90, 4.84)	4.08 (3.03, 5.11)
	5	20%	−0.03 (−0.89, 0.72)	1.17 (0.59, 2.04)	1.23 (0.49, 1.94)
		50%	−0.02 (−1.32, 1.05)	2.99 (1.88, 3.99)	3.08 (1.94, 4.25)
		80%	0.00 (−1.91, 1.61)	4.81 (3.26, 6.19)	4.91 (3.59, 6.36)
		100%	−0.06 (−2.14, 1.98)	5.97 (4.48, 7.74)	6.07 (4.68, 7.78)

Appendix Table 5. The optimal choice for Paxlovid treatment under a range of SARS-CoV-2 transmission scenarios. We estimated the mean (95% confidence interval [CI]) of the number of cases infected averted (millions), number of deaths averted (thousands), number of hospitalizations averted (million), number of courses administered (millions), and net monetary benefit (NMB) in billions of USD, in contrast with baseline, which is scaled to a U.S. population of 328.2 million (*21*) (Appendix Table 6). Each scenario V1-V3 changes one of the base assumptions, as indicated in the second column. Values in the third column are mean and 95% CI in the transmission scenarios (R_e = 1.2 and Treatment rate = 20%) as examples.

Scenarios		R_e = 1.2, Treatment rate = 20%: Incremental net monetary benefits ($ billion), mean (95% CI)
Base	Log relationship between infectiousness and viral load	56.95 (2.62, 122.63)
V1	Log-proportional relationship between infectiousness and viral load+	58.42 (−22.99, 147.37)
V2	Step relationship between infectiousness and viral load&	65.31 (0.71, 141.95)
V3	Sigmoid relationship between infectiousness and viral load*	53.78 (−24.03, 125.87)

+ Infectiousness is proportional log10 of viral load for values above 10^6, as given by log10(Viral load)-6, and is set to zero otherwise (*42*).
& Infectiousness is a constant for viral loads above 10^6, and is set to zero otherwise (*42*).
* Infectiousness has the sigmoid relationship with viral load following the association between viral load and cell culture isolation success rate (*43*).

Appendix Table 6. Dataset

Scenarios	Infectiousness	Measures	R_t	Treatment %	Direct	Indirect	All (Direct + Indirect)
Base	log10	Number of cases averted in U.S. (million)	1.2	20%	−0.22 (−3.82, 1.91)	10.57 (3.03, 21.19)	10.54 (3.03, 21.12)
Base	log10	Number of cases averted in U.S. (million)	1.2	50%	−0.32 (−4.84, 2.37)	25.99 (12.88, 45.11)	25.65 (12.59, 41.19)
Base	log10	Number of cases averted in U.S. (million)	1.2	80%	−0.61 (−5.34, 2.90)	43.13 (25.30, 80.72)	42.58 (25.24, 67.45)
Base	log10	Number of cases averted in U.S. (million)	1.2	100%	−0.84 (−6.19, 3.26)	54.21 (36.77, 81.94)	53.74 (36.67, 81.88)
Base	log10	Number of cases averted in U.S. (million)	1.5	20%	−0.28 (−2.14, 0.99)	4.92 (0.07, 9.32)	4.85 (−0.03, 9.29)
Base	log10	Number of cases averted in U.S. (million)	1.5	50%	−0.44 (−3.69, 1.98)	13.76 (7.55, 19.54)	13.55 (7.51, 19.21)
Base	log10	Number of cases averted in U.S. (million)	1.5	80%	−0.76 (−4.68, 2.54)	23.71 (16.38, 30.21)	23.43 (16.21, 29.92)
Base	log10	Number of cases averted in U.S. (million)	1.5	100%	−0.84 (−4.61, 2.17)	31.33 (23.43, 41.35)	30.84 (23.39, 40.69)
Base	log10	Number of cases averted in U.S. (million)	1.7	20%	−0.13 (−2.14, 1.45)	4.26 (0.07, 8.37)	4.25 (0.00, 8.30)
Base	log10	Number of cases averted in U.S. (million)	1.7	50%	−0.33 (−2.64, 1.91)	10.87 (6.16, 16.70)	10.65 (5.77, 16.70)
Base	log10	Number of cases averted in U.S. (million)	1.7	80%	−0.58 (−2.73, 1.52)	18.39 (10.54, 24.41)	18.15 (10.51, 24.09)
Base	log10	Number of cases averted in U.S. (million)	1.7	100%	−0.57 (−2.73, 1.32)	23.87 (16.47, 30.11)	23.50 (15.85, 29.98)
Base	log10	Number of cases averted in U.S. (million)	2	20%	−0.10 (−1.65, 1.55)	2.93 (−0.23, 6.26)	2.86 (−0.26, 6.16)
Base	log10	Number of cases averted in U.S. (million)	2	50%	−0.39 (−1.94, 1.05)	7.14 (3.03, 11.66)	6.96 (3.00, 11.73)
Base	log10	Number of cases averted in U.S. (million)	2	80%	−0.71 (−2.77, 0.82)	12.01 (7.15, 18.62)	11.63 (6.89, 18.48)
Base	log10	Number of cases averted in U.S. (million)	2	100%	−0.86 (−3.00, 1.19)	15.55 (11.27, 21.38)	15.19 (10.81, 20.99)
Base	log10	Number of cases averted in U.S. (million)	3	20%	−0.05 (−0.49, 0.26)	0.71 (−0.07, 1.45)	0.67 (−0.13, 1.45)
Base	log10	Number of cases averted in U.S. (million)	3	50%	−0.13 (−0.76, 0.40)	1.74 (0.79, 2.83)	1.68 (0.79, 2.77)
Base	log10	Number of cases averted in U.S. (million)	3	80%	−0.21 (−0.82, 0.43)	2.92 (1.94, 4.18)	2.80 (1.78, 4.02)
Base	log10	Number of cases averted in U.S. (million)	3	100%	−0.24 (−0.79, 0.46)	3.77 (2.67, 5.07)	3.67 (2.67, 5.01)
Base	log10	Number of cases averted in U.S. (million)	5	20%	0.00 (−0.07, 0.03)	0.03 (−0.10, 0.13)	0.03 (−0.07, 0.13)
Base	log10	Number of cases averted in U.S. (million)	5	50%	0.00 (−0.10, 0.10)	0.08 (−0.07, 0.20)	0.07 (−0.07, 0.20)

Scenarios	Infectiousness	Measures	R_t	Treatment %	Direct	Indirect	All (Direct + Indirect)
Base	log10	Number of cases averted in U.S. (million)	5	80%	−0.01 (−0.10, 0.07)	0.11 (−0.07, 0.33)	0.11 (−0.07, 0.30)
Base	log10	Number of cases averted in U.S. (million)	5	100%	−0.02 (−0.13, 0.10)	0.14 (−0.07, 0.33)	0.12 (−0.07, 0.33)
Base	log10	Deaths reduced (thousand)	1.2	20%	16.47 (−19.47, 48.11)	19.46 (−14.14, 58.52)	33.85 (1.69, 71.15)
Base	log10	Deaths reduced (thousand)	1.2	50%	43.36 (1.58, 91.31)	48.14 (−8.82, 117.86)	79.11 (35.78, 146.51)
Base	log10	Deaths reduced (thousand)	1.2	80%	65.25 (17.88, 121.49)	76.95 (18.23, 148.21)	113.96 (53.68, 166.28)
Base	log10	Deaths reduced (thousand)	1.2	100%	81.27 (17.71, 143.13)	95.19 (20.40, 165.92)	133.31 (60.98, 185.92)
Base	log10	Deaths reduced (thousand)	1.5	20%	36.87 (−13.91, 86.89)	13.07 (−49.99, 78.46)	50.23 (−1.93, 114.14)
Base	log10	Deaths reduced (thousand)	1.5	50%	94.77 (5.56, 164.45)	35.75 (−48.24, 121.17)	123.79 (39.24, 201.48)
Base	log10	Deaths reduced (thousand)	1.5	80%	150.07 (66.01, 238.88)	61.77 (−71.70, 162.12)	188.19 (89.04, 277.98)
Base	log10	Deaths reduced (thousand)	1.5	100%	186.98 (83.38, 276.20)	82.50 (−48.31, 178.02)	231.40 (124.93, 335.46)
Base	log10	Deaths reduced (thousand)	1.7	20%	48.49 (7.54, 104.72)	11.93 (−44.68, 78.58)	59.43 (9.13, 129.86)
Base	log10	Deaths reduced (thousand)	1.7	50%	120.77 (28.96, 197.79)	35.10 (−62.19, 134.72)	145.44 (45.60, 221.34)
Base	log10	Deaths reduced (thousand)	1.7	80%	190.28 (94.44, 297.07)	52.69 (−43.96, 174.65)	221.79 (115.52, 315.25)
Base	log10	Deaths reduced (thousand)	1.7	100%	238.48 (136.21, 349.11)	70.01 (−40.05, 211.69)	272.44 (180.06, 392.08)
Base	log10	Deaths reduced (thousand)	2	20%	61.73 (5.45, 117.52)	9.02 (−78.52, 74.64)	70.75 (4.05, 144.58)
Base	log10	Deaths reduced (thousand)	2	50%	153.74 (64.08, 240.41)	21.92 (−76.91, 129.39)	174.36 (83.69, 271.33)
Base	log10	Deaths reduced (thousand)	2	80%	244.56 (108.42, 341.85)	44.70 (−75.05, 166.76)	275.73 (158.47, 377.52)
Base	log10	Deaths reduced (thousand)	2	100%	303.84 (158.23, 408.09)	57.57 (−94.86, 173.98)	338.93 (209.58, 456.58)
Base	log10	Deaths reduced (thousand)	3	20%	103.03 (41.24, 174.82)	7.76 (−109.69, 76.28)	109.67 (35.95, 179.83)
Base	log10	Deaths reduced (thousand)	3	50%	257.11 (148.62, 362.85)	16.97 (−91.08, 123.79)	266.69 (156.71, 362.77)
Base	log10	Deaths reduced (thousand)	3	80%	404.90 (266.90, 515.39)	31.25 (−113.81, 158.07)	425.24 (277.20, 552.68)
Base	log10	Deaths reduced (thousand)	3	100%	507.09 (359.15, 646.19)	41.88 (−109.69, 182.87)	525.51 (384.94, 663.72)
Base	log10	Deaths reduced (thousand)	5	20%	154.32 (66.30, 259.38)	0.90 (−120.85, 116.60)	161.81 (56.01, 261.83)
Base	log10	Deaths reduced (thousand)	5	50%	387.76 (231.08, 547.49)	−0.16 (−175.10, 154.01)	399.03 (246.21, 562.19)
Base	log10	Deaths reduced (thousand)	5	80%	620.23 (444.13, 801.75)	2.05 (−263.34, 205.53)	632.56 (447.52, 812.49)
Base	log10	Deaths reduced (thousand)	5	100%	768.62 (569.70, 997.59)	−6.88 (−310.29, 245.90)	782.03 (586.95, 1002.60)
Base	log10	NMB ($ billion)	1.2	20%	25.35 (−35.19, 84.22)	31.17 (−32.77, 103.74)	56.95 (2.62, 122.63)

Scenarios	Infectiousness	Measures	R_t	Treatment %	Direct	Indirect	All (Direct + Indirect)
Base	log10	NMB ($ billion)	1.2	50%	68.03 (−6.92, 151.96)	80.36 (−24.60, 205.73)	135.60 (62.52, 261.32)
Base	log10	NMB ($ billion)	1.2	80%	102.12 (13.07, 189.75)	130.70 (26.22, 254.08)	197.15 (97.62, 293.82)
Base	log10	NMB ($ billion)	1.2	100%	126.96 (16.21, 228.25)	163.44 (22.57, 288.36)	232.26 (107.17, 332.85)
Base	log10	NMB ($ billion)	1.5	20%	58.18 (−19.35, 127.21)	15.55 (−90.17, 134.55)	81.07 (−10.22, 194.06)
Base	log10	NMB ($ billion)	1.5	50%	149.22 (−1.33, 276.58)	45.58 (−99.51, 193.46)	201.38 (50.16, 327.12)
Base	log10	NMB ($ billion)	1.5	80%	238.03 (85.13, 389.40)	83.46 (−140.08, 282.27)	307.42 (125.44, 449.21)
Base	log10	NMB ($ billion)	1.5	100%	295.70 (111.03, 447.24)	115.04 (−99.49, 299.60)	378.72 (198.82, 560.47)
Base	log10	NMB ($ billion)	1.7	20%	76.14 (5.94, 157.33)	12.67 (−82.30, 133.89)	95.66 (8.54, 196.23)
Base	log10	NMB ($ billion)	1.7	50%	190.12 (32.17, 332.79)	39.00 (−130.16, 170.20)	232.35 (80.45, 379.51)
Base	log10	NMB ($ billion)	1.7	80%	300.90 (109.99, 451.75)	60.66 (−112.13, 257.44)	356.40 (176.38, 499.36)
Base	log10	NMB ($ billion)	1.7	100%	377.20 (196.17, 547.91)	86.49 (−102.48, 328.03)	439.82 (266.32, 610.84)
Base	log10	NMB ($ billion)	2	20%	96.74 (3.28, 194.97)	4.57 (−122.98, 126.50)	111.39 (4.57, 246.24)
Base	log10	NMB ($ billion)	2	50%	240.68 (78.62, 396.23)	14.18 (−169.44, 191.31)	276.52 (97.96, 459.36)
Base	log10	NMB ($ billion)	2	80%	385.48 (139.63, 559.60)	41.73 (−184.27, 232.06)	439.08 (208.27, 618.77)
Base	log10	NMB ($ billion)	2	100%	478.73 (205.19, 687.67)	54.86 (−194.98, 282.01)	539.73 (309.83, 764.58)
Base	log10	NMB ($ billion)	3	20%	159.56 (62.97, 284.89)	−2.66 (−172.88, 125.58)	170.17 (60.49, 286.14)
Base	log10	NMB ($ billion)	3	50%	401.32 (219.88, 578.78)	−4.31 (−179.49, 193.58)	417.18 (208.34, 580.13)
Base	log10	NMB ($ billion)	3	80%	633.73 (384.92, 820.60)	1.64 (−239.00, 234.46)	665.92 (414.82, 878.32)
Base	log10	NMB ($ billion)	3	100%	789.52 (541.89, 982.78)	5.43 (−272.37, 270.19)	821.12 (595.37, 1059.33)
Base	log10	NMB ($ billion)	5	20%	234.19 (101.47, 417.79)	−24.98 (−212.90, 142.87)	247.08 (92.89, 409.43)
Base	log10	NMB ($ billion)	5	50%	596.98 (351.54, 838.32)	−53.96 (−348.55, 188.46)	616.81 (366.77, 863.68)
Base	log10	NMB ($ billion)	5	80%	960.35 (646.44, 1267.53)	−77.94 (−500.47, 261.01)	982.74 (685.66, 1298.68)
Base	log10	NMB ($ billion)	5	100%	1189.80 (860.17, 1567.85)	−112.37 (−612.92, 327.29)	1214.09 (885.29, 1605.70)
Base	log10	Treatment courses used (million)	1.2	20%	6.45 (4.78, 7.68)	5.77 (4.38, 7.08)	5.77 (4.38, 7.15)
Base	log10	Treatment courses used (million)	1.2	50%	16.24 (12.55, 18.42)	12.08 (7.31, 14.89)	12.13 (8.86, 14.89)

Scenarios	Infectiousness	Measures	R_t	Treatment %	Direct	Indirect	All (Direct + Indirect)
Base	log10	Treatment courses used (million)	1.2	80%	26.07 (21.35, 29.42)	14.90 (5.93, 20.92)	15.04 (6.79, 21.02)
Base	log10	Treatment courses used (million)	1.2	100%	32.68 (26.66, 36.80)	15.15 (4.94, 21.78)	15.30 (5.93, 21.91)
Base	log10	Treatment courses used (million)	1.5	20%	11.92 (10.84, 12.88)	11.64 (10.31, 12.88)	11.64 (10.35, 12.92)
Base	log10	Treatment courses used (million)	1.5	50%	29.74 (27.51, 31.73)	27.54 (25.60, 29.39)	27.57 (25.57, 29.36)
Base	log10	Treatment courses used (million)	1.5	80%	47.60 (44.88, 50.28)	41.50 (38.68, 44.88)	41.58 (38.78, 45.04)
Base	log10	Treatment courses used (million)	1.5	100%	59.62 (56.41, 63.26)	49.52 (45.17, 53.01)	49.69 (45.34, 52.98)
Base	log10	Treatment courses used (million)	1.7	20%	13.88 (12.62, 15.42)	13.58 (12.42, 15.09)	13.57 (12.42, 15.12)
Base	log10	Treatment courses used (million)	1.7	50%	34.60 (32.55, 36.70)	32.81 (31.04, 34.73)	32.85 (30.87, 34.76)
Base	log10	Treatment courses used (million)	1.7	80%	55.38 (52.09, 58.38)	50.70 (47.61, 53.54)	50.74 (47.61, 53.90)
Base	log10	Treatment courses used (million)	1.7	100%	69.26 (65.30, 73.05)	61.49 (58.02, 63.95)	61.61 (58.22, 64.28)
Base	log10	Treatment courses used (million)	2	20%	16.54 (14.83, 18.09)	16.32 (14.73, 17.96)	16.32 (14.73, 18.02)
Base	log10	Treatment courses used (million)	2	50%	41.40 (39.11, 44.35)	40.03 (37.96, 42.40)	40.07 (37.86, 42.47)
Base	log10	Treatment courses used (million)	2	80%	66.37 (62.57, 70.02)	62.78 (58.68, 66.36)	62.89 (58.58, 66.19)
Base	log10	Treatment courses used (million)	2	100%	83.17 (78.45, 87.68)	77.32 (72.98, 81.94)	77.43 (72.98, 81.81)
Base	log10	Treatment courses used (million)	3	20%	24.58 (22.50, 26.72)	24.42 (22.31, 26.62)	24.41 (22.34, 26.56)
Base	log10	Treatment courses used (million)	3	50%	61.43 (58.45, 64.41)	60.16 (57.00, 63.13)	60.21 (57.07, 63.16)
Base	log10	Treatment courses used (million)	3	80%	98.13 (94.92, 101.78)	94.96 (91.56, 98.71)	95.06 (91.70, 98.68)
Base	log10	Treatment courses used (million)	3	100%	122.84 (119.41, 126.75)	118.06 (114.50, 121.78)	118.21 (114.89, 122.17)
Base	log10	Treatment courses used (million)	5	20%	38.57 (36.08, 41.15)	38.42 (35.98, 41.09)	38.43 (35.98, 41.19)
Base	log10	Treatment courses used (million)	5	50%	96.23 (92.29, 100.16)	95.25 (91.20, 99.27)	95.34 (91.50, 99.34)
Base	log10	Treatment courses used (million)	5	80%	154.18 (150.34, 159.50)	151.64 (146.42, 156.47)	151.84 (146.62, 156.83)
Base	log10	Treatment courses used (million)	5	100%	193.11 (188.47, 198.65)	189.18 (184.81, 194.49)	189.44 (185.27, 195.02)
Base	log10	Hospitalizations reduced (million)	1.2	20%	0.14 (−0.13, 0.40)	0.16 (−0.13, 0.53)	0.28 (0.03, 0.59)
Base	log10	Hospitalizations reduced (million)	1.2	50%	0.36 (0.00, 0.76)	0.41 (−0.07, 0.99)	0.67 (0.33, 1.25)
Base	log10	Hospitalizations reduced (million)	1.2	80%	0.55 (0.10, 0.96)	0.65 (0.16, 1.22)	0.96 (0.49, 1.42)
Base	log10	Hospitalizations reduced (million)	1.2	100%	0.68 (0.20, 1.15)	0.80 (0.16, 1.35)	1.13 (0.56, 1.58)
Base	log10	Hospitalizations reduced (million)	1.5	20%	0.30 (−0.03, 0.63)	0.10 (−0.40, 0.66)	0.41 (−0.03, 0.92)
Base	log10	Hospitalizations reduced (million)	1.5	50%	0.77 (0.07, 1.38)	0.28 (−0.40, 0.96)	1.01 (0.30, 1.58)
Base	log10	Hospitalizations reduced (million)	1.5	80%	1.23 (0.49, 1.91)	0.48 (−0.43, 1.38)	1.53 (0.66, 2.14)
Base	log10	Hospitalizations reduced (million)	1.5	100%	1.53 (0.66, 2.24)	0.65 (−0.36, 1.52)	1.88 (0.99, 2.67)
Base	log10	Hospitalizations reduced (million)	1.7	20%	0.39 (0.07, 0.79)	0.09 (−0.33, 0.66)	0.48 (0.07, 0.92)
Base	log10	Hospitalizations reduced (million)	1.7	50%	0.97 (0.26, 1.68)	0.26 (−0.49, 0.86)	1.16 (0.49, 1.85)

Scenarios	Infectiousness	Measures	R_t	Treatment %	Direct	Indirect	All (Direct + Indirect)
Base	log10	Hospitalizations reduced (million)	1.7	80%	1.54 (0.69, 2.27)	0.40 (−0.43, 1.32)	1.78 (0.89, 2.44)
Base	log10	Hospitalizations reduced (million)	1.7	100%	1.93 (1.15, 2.73)	0.55 (−0.36, 1.61)	2.19 (1.38, 2.93)
Base	log10	Hospitalizations reduced (million)	2	20%	0.49 (0.03, 0.92)	0.06 (−0.46, 0.59)	0.55 (0.10, 1.19)
Base	log10	Hospitalizations reduced (million)	2	50%	1.22 (0.46, 1.94)	0.16 (−0.72, 1.02)	1.38 (0.59, 2.24)
Base	log10	Hospitalizations reduced (million)	2	80%	1.95 (0.86, 2.80)	0.34 (−0.76, 1.19)	2.19 (1.12, 3.00)
Base	log10	Hospitalizations reduced (million)	2	100%	2.43 (1.19, 3.43)	0.44 (−0.69, 1.42)	2.69 (1.65, 3.72)
Base	log10	Hospitalizations reduced (million)	3	20%	0.80 (0.40, 1.32)	0.05 (−0.69, 0.59)	0.85 (0.36, 1.38)
Base	log10	Hospitalizations reduced (million)	3	50%	2.01 (1.15, 2.83)	0.13 (−0.63, 1.12)	2.08 (1.12, 2.83)
Base	log10	Hospitalizations reduced (million)	3	80%	3.17 (2.08, 4.05)	0.24 (−0.82, 1.35)	3.30 (2.14, 4.22)
Base	log10	Hospitalizations reduced (million)	3	100%	3.95 (2.90, 4.84)	0.31 (−0.89, 1.48)	4.08 (3.03, 5.11)
Base	log10	Hospitalizations reduced (million)	5	20%	1.17 (0.59, 2.04)	−0.03 (−0.89, 0.72)	1.23 (0.49, 1.94)
Base	log10	Hospitalizations reduced (million)	5	50%	2.99 (1.88, 3.99)	−0.02 (−1.32, 1.05)	3.08 (1.94, 4.25)
Base	log10	Hospitalizations reduced (million)	5	80%	4.81 (3.26, 6.19)	0.00 (−1.91, 1.61)	4.91 (3.59, 6.36)
Base	log10	Hospitalizations reduced (million)	5	100%	5.97 (4.48, 7.74)	−0.06 (−2.14, 1.98)	6.07 (4.68, 7.78)
V1	log10-proportional	Number of cases averted in U.S. (million)	1.2	20%	−0.23 (−3.29, 3.26)	9.68 (0.33, 23.16)	9.58 (0.49, 23.16)
V1	log10-proportional	Number of cases averted in U.S. (million)	1.2	50%	−0.51 (−6.52, 3.53)	26.14 (11.83, 47.61)	25.91 (11.80, 47.61)
V1	log10-proportional	Number of cases averted in U.S. (million)	1.2	80%	−0.80 (−6.66, 3.89)	43.60 (25.80, 71.83)	43.31 (25.77, 71.63)
V1	log10-proportional	Number of cases averted in U.S. (million)	1.2	100%	−1.23 (−5.67, 2.50)	54.28 (34.96, 81.22)	54.10 (34.93, 81.19)
V1	log10-proportional	Number of cases averted in U.S. (million)	1.5	20%	−0.33 (−3.10, 1.55)	4.77 (−0.43, 10.74)	4.73 (−0.43, 10.74)
V1	log10-proportional	Number of cases averted in U.S. (million)	1.5	50%	−0.60 (−4.12, 2.37)	13.72 (5.83, 21.42)	13.61 (5.77, 22.08)
V1	log10-proportional	Number of cases averted in U.S. (million)	1.5	80%	−1.09 (−5.34, 3.06)	23.27 (14.04, 32.75)	23.18 (13.38, 32.62)
V1	log10-proportional	Number of cases averted in U.S. (million)	1.5	100%	−1.19 (−5.21, 3.49)	30.51 (19.24, 46.92)	30.32 (18.81, 44.22)
V1	log10-proportional	Number of cases averted in U.S. (million)	1.7	20%	−0.03 (−2.90, 2.27)	4.31 (−0.40, 10.48)	4.26 (−0.66, 10.44)
V1	log10-proportional	Number of cases averted in U.S. (million)	1.7	50%	−0.18 (−3.49, 3.99)	10.84 (4.94, 17.76)	10.84 (4.32, 17.73)
V1	log10-proportional	Number of cases averted in U.S. (million)	1.7	80%	−0.44 (−3.99, 3.95)	18.14 (10.38, 25.63)	17.86 (9.56, 25.21)
V1	log10-proportional	Number of cases averted in U.S. (million)	1.7	100%	−0.56 (−4.58, 4.28)	23.47 (14.99, 33.08)	23.25 (14.96, 32.68)
V1	log10-proportional	Number of cases averted in U.S. (million)	2	20%	−0.21 (−2.90, 2.01)	2.97 (−3.16, 9.13)	2.88 (−4.68, 8.04)

Scenarios	Infectiousness	Measures	R_t	Treatment %	Direct	Indirect	All (Direct + Indirect)
V1	log10-proportional	Number of cases averted in U.S. (million)	2	50%	−0.55 (−5.11, 2.24)	7.50 (1.94, 14.00)	7.48 (1.71, 13.97)
V1	log10-proportional	Number of cases averted in U.S. (million)	2	80%	−0.68 (−5.21, 3.23)	12.97 (6.03, 20.53)	12.82 (5.60, 20.53)
V1	log10-proportional	Number of cases averted in U.S. (million)	2	100%	−0.78 (−5.11, 4.15)	16.57 (8.80, 22.73)	16.42 (8.11, 22.50)
V1	log10-proportional	Number of cases averted in U.S. (million)	3	20%	−0.03 (−0.53, 0.63)	0.66 (−0.16, 1.52)	0.65 (−0.16, 1.52)
V1	log10-proportional	Number of cases averted in U.S. (million)	3	50%	−0.11 (−0.82, 0.63)	1.77 (0.63, 2.93)	1.73 (0.63, 3.13)
V1	log10-proportional	Number of cases averted in U.S. (million)	3	80%	−0.23 (−1.12, 0.63)	3.11 (1.94, 4.71)	3.07 (1.71, 4.58)
V1	log10-proportional	Number of cases averted in U.S. (million)	3	100%	−0.27 (−1.12, 0.49)	4.07 (2.77, 5.30)	3.94 (2.50, 5.47)
V1	log10-proportional	Number of cases averted in U.S. (million)	5	20%	0.00 (−0.10, 0.10)	0.04 (−0.10, 0.23)	0.04 (−0.10, 0.23)
V1	log10-proportional	Number of cases averted in U.S. (million)	5	50%	0.00 (−0.13, 0.13)	0.10 (−0.07, 0.30)	0.09 (−0.07, 0.30)
V1	log10-proportional	Number of cases averted in U.S. (million)	5	80%	−0.01 (−0.16, 0.13)	0.15 (−0.07, 0.36)	0.14 (−0.10, 0.36)
V1	log10-proportional	Number of cases averted in U.S. (million)	5	100%	−0.01 (−0.13, 0.16)	0.19 (−0.03, 0.53)	0.17 (−0.03, 0.46)
V1	log10-proportional	Deaths reduced (thousand)	1.2	20%	16.15 (−7.13, 46.43)	19.76 (−33.68, 71.44)	34.03 (−14.27, 81.90)
V1	log10-proportional	Deaths reduced (thousand)	1.2	50%	44.11 (−3.92, 89.28)	51.05 (−26.43, 138.79)	82.35 (7.31, 163.00)
V1	log10-proportional	Deaths reduced (thousand)	1.2	80%	67.64 (8.59, 138.97)	81.04 (−0.60, 160.94)	119.05 (40.86, 189.58)
V1	log10-proportional	Deaths reduced (thousand)	1.2	100%	83.65 (16.44, 151.47)	100.01 (20.42, 171.24)	139.10 (73.01, 212.84)
V1	log10-proportional	Deaths reduced (thousand)	1.5	20%	33.63 (−0.12, 78.28)	12.81 (−42.80, 71.09)	47.99 (6.95, 101.49)
V1	log10-proportional	Deaths reduced (thousand)	1.5	50%	90.69 (36.36, 170.90)	39.94 (−40.40, 130.11)	121.03 (47.64, 208.50)
V1	log10-proportional	Deaths reduced (thousand)	1.5	80%	144.10 (49.36, 230.93)	59.86 (−33.14, 152.94)	180.95 (90.04, 265.45)
V1	log10-proportional	Deaths reduced (thousand)	1.5	100%	182.30 (86.35, 270.41)	79.50 (−26.56, 196.44)	218.48 (123.64, 336.94)
V1	log10-proportional	Deaths reduced (thousand)	1.7	20%	46.15 (−5.03, 89.22)	11.64 (−57.23, 85.24)	57.36 (2.24, 112.79)
V1	log10-proportional	Deaths reduced (thousand)	1.7	50%	114.48 (27.48, 185.66)	31.45 (−43.26, 138.69)	141.60 (70.57, 236.61)
V1	log10-proportional	Deaths reduced (thousand)	1.7	80%	183.45 (76.63, 278.15)	55.14 (−38.76, 176.52)	217.61 (122.43, 320.85)
V1	log10-proportional	Deaths reduced (thousand)	1.7	100%	229.49 (124.71, 338.22)	69.92 (−52.10, 187.19)	263.30 (146.93, 365.55)
V1	log10-proportional	Deaths reduced (thousand)	2	20%	58.54 (9.12, 112.42)	11.68 (−58.97, 88.91)	69.51 (−5.67, 153.17)
V1	log10-proportional	Deaths reduced (thousand)	2	50%	147.20 (59.10, 222.91)	25.51 (−79.17, 119.34)	165.22 (69.98, 249.83)
V1	log10-proportional	Deaths reduced (thousand)	2	80%	235.31 (142.64, 353.27)	50.51 (−68.60, 156.17)	265.32 (167.86, 383.74)

Scenarios	Infectiousness	Measures	R_t	Treatment %	Direct	Indirect	All (Direct + Indirect)
V1	log10-proportional	Deaths reduced (thousand)	2	100%	291.76 (176.58, 422.76)	63.44 (−74.48, 184.51)	324.68 (203.59, 456.88)
V1	log10-proportional	Deaths reduced (thousand)	3	20%	102.22 (33.69, 176.18)	14.81 (−88.66, 110.68)	111.41 (39.11, 199.14)
V1	log10-proportional	Deaths reduced (thousand)	3	50%	252.10 (156.92, 352.38)	24.51 (−104.53, 150.04)	267.64 (160.55, 374.07)
V1	log10-proportional	Deaths reduced (thousand)	3	80%	399.96 (250.34, 527.24)	34.29 (−153.84, 174.53)	418.93 (271.57, 551.23)
V1	log10-proportional	Deaths reduced (thousand)	3	100%	498.05 (369.00, 636.06)	41.63 (−133.15, 202.59)	516.84 (399.85, 663.90)
V1	log10-proportional	Deaths reduced (thousand)	5	20%	143.09 (44.85, 221.24)	1.03 (−102.17, 106.06)	151.24 (28.65, 246.44)
V1	log10-proportional	Deaths reduced (thousand)	5	50%	363.16 (205.05, 492.13)	−1.54 (−176.25, 185.46)	379.90 (240.41, 509.77)
V1	log10-proportional	Deaths reduced (thousand)	5	80%	591.48 (414.50, 772.95)	−0.61 (−259.31, 225.62)	607.46 (410.58, 780.11)
V1	log10-proportional	Deaths reduced (thousand)	5	100%	744.21 (555.56, 948.46)	2.30 (−204.90, 238.01)	762.27 (578.26, 948.34)
V1	log10-proportional	NMB ($ billion)	1.2	20%	26.38 (−17.97, 77.89)	32.56 (−53.16, 132.75)	58.42 (−22.99, 147.37)
V1	log10-proportional	NMB ($ billion)	1.2	50%	71.10 (−21.67, 144.25)	85.19 (−58.30, 226.41)	141.49 (11.22, 272.68)
V1	log10-proportional	NMB ($ billion)	1.2	80%	108.00 (−5.39, 211.17)	137.99 (−16.62, 268.51)	206.76 (68.35, 340.23)
V1	log10-proportional	NMB ($ billion)	1.2	100%	133.23 (11.65, 249.42)	172.64 (34.64, 295.28)	244.01 (123.06, 388.28)
V1	log10-proportional	NMB ($ billion)	1.5	20%	54.78 (−10.38, 134.97)	18.11 (−78.60, 116.73)	79.85 (−2.13, 175.76)
V1	log10-proportional	NMB ($ billion)	1.5	50%	145.43 (54.07, 266.83)	56.00 (−94.11, 208.78)	198.98 (70.70, 341.65)
V1	log10-proportional	NMB ($ billion)	1.5	80%	232.33 (88.94, 391.27)	86.58 (−63.72, 256.86)	299.60 (163.65, 448.54)
V1	log10-proportional	NMB ($ billion)	1.5	100%	293.67 (140.23, 451.41)	116.19 (−61.40, 324.23)	361.50 (213.47, 546.30)
V1	log10-proportional	NMB ($ billion)	1.7	20%	74.18 (−6.97, 149.06)	12.39 (−115.95, 143.27)	92.47 (−8.09, 189.80)
V1	log10-proportional	NMB ($ billion)	1.7	50%	183.32 (49.23, 333.69)	36.96 (−107.80, 223.19)	229.69 (112.26, 409.15)
V1	log10-proportional	NMB ($ billion)	1.7	80%	293.23 (118.15, 476.10)	68.72 (−100.37, 270.10)	352.17 (187.33, 534.45)
V1	log10-proportional	NMB ($ billion)	1.7	100%	366.16 (178.21, 548.88)	86.87 (−129.16, 307.14)	426.63 (235.62, 631.03)
V1	log10-proportional	NMB ($ billion)	2	20%	92.71 (1.97, 189.01)	11.32 (−130.04, 134.33)	111.16 (−23.52, 239.59)
V1	log10-proportional	NMB ($ billion)	2	50%	235.13 (84.42, 361.11)	24.31 (−148.18, 191.67)	265.87 (116.58, 411.46)
V1	log10-proportional	NMB ($ billion)	2	80%	375.17 (217.96, 572.52)	56.23 (−155.61, 219.98)	427.06 (264.40, 645.71)

Scenarios	Infectiousness	Measures	R_t	Treatment %	Direct	Indirect	All (Direct + Indirect)
V1	log10-proportional	NMB ($ billion)	2	100%	463.52 (275.26, 697.98)	69.58 (−172.78, 280.70)	521.94 (320.73, 766.65)
V1	log10-proportional	NMB ($ billion)	3	20%	158.56 (37.55, 261.16)	9.71 (−164.07, 176.62)	173.43 (54.85, 315.51)
V1	log10-proportional	NMB ($ billion)	3	50%	394.79 (228.36, 542.75)	12.37 (−207.26, 203.07)	421.14 (246.16, 592.96)
V1	log10-proportional	NMB ($ billion)	3	80%	625.68 (373.94, 844.12)	12.41 (−292.61, 228.89)	659.71 (409.27, 872.60)
V1	log10-proportional	NMB ($ billion)	3	100%	777.18 (546.23, 994.81)	12.37 (−265.89, 309.20)	812.41 (620.29, 1015.68)
V1	log10-proportional	NMB ($ billion)	5	20%	217.41 (59.78, 366.05)	−22.84 (−235.77, 151.93)	232.36 (36.11, 383.17)
V1	log10-proportional	NMB ($ billion)	5	50%	557.85 (316.19, 751.54)	−55.98 (−363.93, 206.17)	586.89 (336.42, 818.34)
V1	log10-proportional	NMB ($ billion)	5	80%	912.76 (627.53, 1197.26)	−81.56 (−497.01, 293.76)	942.06 (623.17, 1215.65)
V1	log10-proportional	NMB ($ billion)	5	100%	1147.82 (789.06, 1496.34)	−97.63 (−447.29, 279.14)	1181.88 (858.51, 1509.11)
V1	log10-proportional	Treatment courses used (million)	1.2	20%	6.54 (5.21, 7.91)	5.93 (4.35, 7.15)	5.94 (4.35, 7.15)
V1	log10-proportional	Treatment courses used (million)	1.2	50%	16.47 (13.38, 19.01)	12.36 (8.86, 15.49)	12.39 (8.86, 15.49)
V1	log10-proportional	Treatment courses used (million)	1.2	80%	26.37 (21.61, 29.95)	15.13 (7.55, 20.56)	15.20 (7.55, 20.69)
V1	log10-proportional	Treatment courses used (million)	1.2	100%	33.17 (27.45, 37.63)	15.41 (6.69, 21.58)	15.47 (6.72, 21.65)
V1	log10-proportional	Treatment courses used (million)	1.5	20%	11.27 (9.88, 12.65)	10.92 (9.75, 12.36)	10.92 (9.79, 12.36)
V1	log10-proportional	Treatment courses used (million)	1.5	50%	28.15 (25.77, 30.71)	25.97 (23.76, 28.43)	25.98 (23.76, 28.43)
V1	log10-proportional	Treatment courses used (million)	1.5	80%	45.11 (42.40, 48.93)	39.19 (36.54, 42.57)	39.22 (36.38, 42.60)
V1	log10-proportional	Treatment courses used (million)	1.5	100%	56.46 (53.08, 60.56)	46.80 (43.00, 50.58)	46.88 (43.03, 50.58)
V1	log10-proportional	Treatment courses used (million)	1.7	20%	13.33 (11.83, 14.63)	13.06 (11.80, 14.50)	13.06 (11.80, 14.56)
V1	log10-proportional	Treatment courses used (million)	1.7	50%	33.29 (29.88, 35.58)	31.62 (29.09, 34.33)	31.61 (29.16, 34.10)
V1	log10-proportional	Treatment courses used (million)	1.7	80%	53.37 (49.62, 56.64)	48.76 (45.57, 51.73)	48.83 (45.73, 51.89)
V1	log10-proportional	Treatment courses used (million)	1.7	100%	66.72 (63.13, 70.11)	59.43 (55.88, 63.36)	59.50 (55.81, 63.39)
V1	log10-proportional	Treatment courses used (million)	2	20%	15.74 (14.23, 17.27)	15.53 (14.17, 17.00)	15.54 (14.17, 17.17)
V1	log10-proportional	Treatment courses used (million)	2	50%	39.47 (36.77, 43.13)	38.05 (35.45, 40.69)	38.04 (35.42, 40.79)
V1	log10-proportional	Treatment courses used (million)	2	80%	63.19 (58.81, 67.18)	59.51 (56.47, 62.47)	59.56 (56.37, 62.47)
V1	log10-proportional	Treatment courses used (million)	2	100%	78.97 (74.00, 84.32)	73.24 (69.36, 77.36)	73.29 (69.72, 77.13)
V1	log10-proportional	Treatment courses used (million)	3	20%	24.12 (22.21, 25.73)	23.90 (22.14, 25.67)	23.91 (22.21, 25.67)
V1	log10-proportional	Treatment courses used (million)	3	50%	60.41 (57.53, 63.56)	59.31 (56.34, 62.31)	59.33 (56.34, 62.37)
V1	log10-proportional	Treatment courses used (million)	3	80%	96.70 (92.78, 100.00)	93.67 (90.51, 96.74)	93.71 (90.58, 96.84)
V1	log10-proportional	Treatment courses used (million)	3	100%	121.11 (117.43, 125.24)	116.26 (112.88, 120.33)	116.41 (112.91, 120.43)

Scenarios	Infectiousness	Measures	R_t	Treatment %	Direct	Indirect	All (Direct + Indirect)
V1	log10-proportional	Treatment courses used (million)	5	20%	37.34 (35.06, 40.33)	37.15 (35.16, 39.83)	37.19 (35.32, 39.90)
V1	log10-proportional	Treatment courses used (million)	5	50%	93.34 (89.85, 97.10)	92.18 (88.73, 96.08)	92.29 (88.73, 96.08)
V1	log10-proportional	Treatment courses used (million)	5	80%	149.39 (145.50, 153.57)	146.60 (142.83, 150.25)	146.78 (143.36, 150.48)
V1	log10-proportional	Treatment courses used (million)	5	100%	186.99 (182.30, 191.86)	182.61 (177.86, 186.36)	182.85 (177.89, 186.82)
V1	log10-proportional	Hospitalizations reduced (million)	1.2	20%	0.14 (−0.07, 0.40)	0.16 (−0.26, 0.63)	0.29 (−0.10, 0.69)
V1	log10-proportional	Hospitalizations reduced (million)	1.2	50%	0.38 (−0.07, 0.76)	0.43 (−0.30, 1.05)	0.69 (0.10, 1.28)
V1	log10-proportional	Hospitalizations reduced (million)	1.2	80%	0.58 (0.00, 1.05)	0.68 (−0.03, 1.28)	1.00 (0.33, 1.61)
V1	log10-proportional	Hospitalizations reduced (million)	1.2	100%	0.71 (0.16, 1.28)	0.84 (0.20, 1.38)	1.18 (0.63, 1.88)
V1	log10-proportional	Hospitalizations reduced (million)	1.5	20%	0.29 (0.00, 0.63)	0.11 (−0.33, 0.56)	0.40 (0.00, 0.86)
V1	log10-proportional	Hospitalizations reduced (million)	1.5	50%	0.75 (0.33, 1.32)	0.32 (−0.36, 1.02)	0.99 (0.36, 1.65)
V1	log10-proportional	Hospitalizations reduced (million)	1.5	80%	1.20 (0.59, 1.91)	0.50 (−0.20, 1.32)	1.50 (0.86, 2.21)
V1	log10-proportional	Hospitalizations reduced (million)	1.5	100%	1.51 (0.82, 2.31)	0.66 (−0.20, 1.65)	1.80 (1.09, 2.64)
V1	log10-proportional	Hospitalizations reduced (million)	1.7	20%	0.38 (0.00, 0.72)	0.09 (−0.49, 0.69)	0.46 (0.00, 0.96)
V1	log10-proportional	Hospitalizations reduced (million)	1.7	50%	0.94 (0.30, 1.61)	0.25 (−0.46, 1.09)	1.14 (0.59, 2.04)
V1	log10-proportional	Hospitalizations reduced (million)	1.7	80%	1.50 (0.66, 2.31)	0.43 (−0.40, 1.38)	1.75 (1.05, 2.57)
V1	log10-proportional	Hospitalizations reduced (million)	1.7	100%	1.87 (0.99, 2.77)	0.54 (−0.49, 1.52)	2.12 (1.25, 3.03)
V1	log10-proportional	Hospitalizations reduced (million)	2	20%	0.47 (0.03, 0.89)	0.09 (−0.56, 0.66)	0.55 (−0.03, 1.15)
V1	log10-proportional	Hospitalizations reduced (million)	2	50%	1.19 (0.49, 1.78)	0.21 (−0.56, 0.96)	1.33 (0.66, 2.01)
V1	log10-proportional	Hospitalizations reduced (million)	2	80%	1.90 (1.15, 2.77)	0.41 (−0.63, 1.19)	2.13 (1.38, 3.16)
V1	log10-proportional	Hospitalizations reduced (million)	2	100%	2.35 (1.48, 3.43)	0.49 (−0.59, 1.52)	2.60 (1.65, 3.76)
V1	log10-proportional	Hospitalizations reduced (million)	3	20%	0.79 (0.23, 1.25)	0.10 (−0.69, 0.89)	0.86 (0.30, 1.48)
V1	log10-proportional	Hospitalizations reduced (million)	3	50%	1.98 (1.22, 2.70)	0.21 (−0.86, 1.09)	2.09 (1.28, 2.87)
V1	log10-proportional	Hospitalizations reduced (million)	3	80%	3.13 (1.98, 4.09)	0.29 (−1.12, 1.28)	3.28 (2.14, 4.25)
V1	log10-proportional	Hospitalizations reduced (million)	3	100%	3.89 (2.83, 4.88)	0.35 (−0.92, 1.65)	4.04 (3.10, 5.04)
V1	log10-proportional	Hospitalizations reduced (million)	5	20%	1.09 (0.40, 1.78)	−0.02 (−1.02, 0.72)	1.16 (0.26, 1.88)
V1	log10-proportional	Hospitalizations reduced (million)	5	50%	2.81 (1.65, 3.76)	−0.04 (−1.42, 1.22)	2.94 (1.85, 3.99)
V1	log10-proportional	Hospitalizations reduced (million)	5	80%	4.58 (3.23, 5.90)	−0.03 (−1.91, 1.61)	4.71 (3.26, 5.96)
V1	log10-proportional	Hospitalizations reduced (million)	5	100%	5.76 (4.18, 7.35)	−0.01 (−1.55, 1.68)	5.91 (4.45, 7.41)
V2	threshold	Number of cases averted in U.S. (million)	1.2	20%	−0.30 (−3.06, 1.94)	11.37 (3.66, 26.39)	11.27 (3.66, 26.36)
V2	threshold	Number of cases averted in U.S. (million)	1.2	50%	−0.70 (−5.50, 2.47)	29.90 (17.30, 45.14)	29.48 (17.23, 45.11)
V2	threshold	Number of cases averted in U.S. (million)	1.2	80%	−1.00 (−6.66, 2.60)	47.54 (33.15, 69.62)	47.10 (32.62, 69.62)

Scenarios	Infectiousness	Measures	R_t	Treatment %	Direct	Indirect	All (Direct + Indirect)
V2	threshold	Number of cases averted in U.S. (million)	1.2	100%	−1.31 (−6.66, 3.26)	59.91 (43.89, 81.05)	59.33 (43.13, 78.29)
V2	threshold	Number of cases averted in U.S. (million)	1.5	20%	−0.18 (−2.04, 1.65)	5.84 (0.96, 10.48)	5.73 (0.72, 10.44)
V2	threshold	Number of cases averted in U.S. (million)	1.5	50%	−0.40 (−2.73, 1.78)	16.29 (10.94, 23.99)	16.11 (10.94, 24.22)
V2	threshold	Number of cases averted in U.S. (million)	1.5	80%	−0.63 (−3.03, 2.08)	28.52 (20.82, 36.84)	28.14 (20.46, 36.14)
V2	threshold	Number of cases averted in U.S. (million)	1.5	100%	−0.74 (−3.03, 2.08)	38.71 (30.31, 48.93)	38.28 (30.21, 49.29)
V2	threshold	Number of cases averted in U.S. (million)	1.7	20%	0.02 (−1.09, 1.35)	4.87 (0.53, 8.50)	4.76 (0.49, 8.50)
V2	threshold	Number of cases averted in U.S. (million)	1.7	50%	−0.27 (−2.67, 1.85)	12.75 (8.57, 18.65)	12.44 (7.58, 18.48)
V2	threshold	Number of cases averted in U.S. (million)	1.7	80%	−0.51 (−2.73, 1.75)	21.96 (16.31, 27.15)	21.59 (15.85, 27.05)
V2	threshold	Number of cases averted in U.S. (million)	1.7	100%	−0.69 (−3.06, 1.61)	28.51 (21.55, 34.66)	28.16 (20.56, 34.40)
V2	threshold	Number of cases averted in U.S. (million)	2	20%	−0.14 (−1.75, 1.22)	3.25 (0.20, 6.23)	3.15 (−0.13, 6.00)
V2	threshold	Number of cases averted in U.S. (million)	2	50%	−0.39 (−2.08, 1.68)	8.25 (4.61, 12.32)	8.09 (4.35, 12.03)
V2	threshold	Number of cases averted in U.S. (million)	2	80%	−0.62 (−2.97, 1.55)	14.28 (9.39, 18.58)	13.99 (9.36, 17.89)
V2	threshold	Number of cases averted in U.S. (million)	2	100%	−0.76 (−3.79, 2.77)	18.89 (13.34, 24.38)	18.56 (12.72, 23.66)
V2	threshold	Number of cases averted in U.S. (million)	3	20%	−0.03 (−0.40, 0.23)	0.77 (0.16, 1.45)	0.75 (0.10, 1.35)
V2	threshold	Number of cases averted in U.S. (million)	3	50%	−0.11 (−0.53, 0.26)	2.02 (1.15, 2.97)	1.94 (1.12, 2.87)
V2	threshold	Number of cases averted in U.S. (million)	3	80%	−0.17 (−0.72, 0.43)	3.39 (2.08, 4.45)	3.29 (2.14, 4.45)
V2	threshold	Number of cases averted in U.S. (million)	3	100%	−0.22 (−0.79, 0.30)	4.37 (3.10, 5.67)	4.23 (2.87, 5.63)
V2	threshold	Number of cases averted in U.S. (million)	5	20%	0.00 (−0.07, 0.07)	0.03 (−0.07, 0.13)	0.03 (−0.07, 0.13)
V2	threshold	Number of cases averted in U.S. (million)	5	50%	0.00 (−0.10, 0.07)	0.09 (−0.07, 0.23)	0.08 (−0.07, 0.23)
V2	threshold	Number of cases averted in U.S. (million)	5	80%	−0.01 (−0.16, 0.13)	0.14 (−0.07, 0.36)	0.12 (−0.07, 0.33)
V2	threshold	Number of cases averted in U.S. (million)	5	100%	−0.02 (−0.16, 0.10)	0.17 (−0.03, 0.40)	0.16 (−0.07, 0.40)
V2	threshold	Deaths reduced (thousand)	1.2	20%	17.15 (−5.44, 39.23)	23.64 (−19.82, 67.70)	38.07 (−1.81, 78.46)
V2	threshold	Deaths reduced (thousand)	1.2	50%	43.42 (−1.53, 82.14)	59.43 (0.59, 119.73)	88.79 (32.68, 139.21)

Scenarios	Infectiousness	Measures	R_t	Treatment %	Direct	Indirect	All (Direct + Indirect)
V2	threshold	Deaths reduced (thousand)	1.2	80%	69.08 (14.55, 125.17)	91.36 (21.40, 153.53)	125.88 (60.56, 182.18)
V2	threshold	Deaths reduced (thousand)	1.2	100%	85.50 (28.36, 141.43)	112.59 (39.52, 178.55)	145.22 (82.37, 205.39)
V2	threshold	Deaths reduced (thousand)	1.5	20%	38.90 (−3.80, 89.05)	20.64 (−39.52, 74.91)	55.65 (9.18, 108.35)
V2	threshold	Deaths reduced (thousand)	1.5	50%	97.29 (34.09, 173.00)	45.62 (−40.74, 129.39)	132.70 (69.69, 219.13)
V2	threshold	Deaths reduced (thousand)	1.5	80%	154.81 (71.13, 245.44)	79.46 (−46.08, 176.96)	200.81 (97.89, 285.56)
V2	threshold	Deaths reduced (thousand)	1.5	100%	189.57 (96.72, 283.47)	105.21 (−7.20, 192.23)	243.83 (128.26, 332.42)
V2	threshold	Deaths reduced (thousand)	1.7	20%	48.21 (−0.12, 93.07)	12.45 (−51.97, 65.31)	57.75 (−6.85, 114.48)
V2	threshold	Deaths reduced (thousand)	1.7	50%	119.90 (30.23, 204.81)	34.10 (−80.21, 115.88)	143.57 (40.77, 231.89)
V2	threshold	Deaths reduced (thousand)	1.7	80%	192.31 (103.54, 292.20)	66.60 (−53.11, 158.34)	228.64 (125.36, 318.28)
V2	threshold	Deaths reduced (thousand)	1.7	100%	236.97 (149.69, 321.74)	84.82 (−29.04, 191.11)	278.39 (186.82, 362.48)
V2	threshold	Deaths reduced (thousand)	2	20%	60.91 (1.93, 115.54)	11.41 (−80.14, 81.83)	71.67 (−8.76, 140.83)
V2	threshold	Deaths reduced (thousand)	2	50%	150.46 (46.38, 231.93)	28.97 (−113.92, 112.25)	174.79 (44.59, 261.64)
V2	threshold	Deaths reduced (thousand)	2	80%	241.23 (117.19, 325.13)	54.42 (−91.38, 161.98)	275.81 (152.71, 365.84)
V2	threshold	Deaths reduced (thousand)	2	100%	304.10 (171.89, 402.99)	72.76 (−66.30, 176.92)	342.22 (201.93, 444.01)
V2	threshold	Deaths reduced (thousand)	3	20%	100.07 (32.03, 174.68)	10.84 (−99.57, 119.45)	111.08 (14.27, 205.09)
V2	threshold	Deaths reduced (thousand)	3	50%	249.78 (133.48, 354.23)	23.75 (−100.42, 187.99)	272.99 (161.82, 389.79)
V2	threshold	Deaths reduced (thousand)	3	80%	400.58 (282.98, 533.51)	37.29 (−107.29, 254.93)	427.53 (307.19, 580.04)
V2	threshold	Deaths reduced (thousand)	3	100%	503.61 (361.84, 667.05)	52.91 (−96.46, 262.92)	531.16 (388.16, 675.35)
V2	threshold	Deaths reduced (thousand)	5	20%	154.99 (67.61, 247.44)	−2.63 (−108.52, 97.96)	156.35 (44.86, 243.04)
V2	threshold	Deaths reduced (thousand)	5	50%	382.55 (239.73, 524.66)	−3.43 (−171.40, 144.47)	387.41 (246.14, 530.07)
V2	threshold	Deaths reduced (thousand)	5	80%	609.96 (449.45, 814.46)	5.58 (−194.93, 261.04)	623.02 (465.10, 822.44)
V2	threshold	Deaths reduced (thousand)	5	100%	753.47 (585.00, 982.33)	−0.19 (−219.71, 291.34)	772.75 (604.30, 995.18)
V2	threshold	NMB ($ billion)	1.2	20%	27.40 (−16.31, 68.82)	39.29 (−41.89, 112.38)	65.31 (0.71, 141.95)
V2	threshold	NMB ($ billion)	1.2	50%	68.75 (1.74, 141.86)	100.42 (−4.40, 200.97)	153.23 (55.32, 240.08)
V2	threshold	NMB ($ billion)	1.2	80%	110.58 (23.88, 203.74)	157.30 (29.39, 261.71)	219.81 (102.90, 308.62)
V2	threshold	NMB ($ billion)	1.2	100%	135.48 (32.55, 244.15)	195.35 (71.31, 299.55)	254.70 (151.88, 358.54)

Scenarios	Infectiousness	Measures	R_t	Treatment %	Direct	Indirect	All (Direct + Indirect)
V2	threshold	NMB ($ billion)	1.5	20%	62.02 (−24.68, 135.07)	28.64 (−68.19, 123.77)	90.37 (12.18, 177.46)
V2	threshold	NMB ($ billion)	1.5	50%	154.30 (39.73, 283.09)	63.29 (−82.45, 206.15)	216.02 (106.80, 373.11)
V2	threshold	NMB ($ billion)	1.5	80%	246.14 (94.47, 398.27)	116.48 (−98.95, 294.35)	329.44 (149.48, 505.84)
V2	threshold	NMB ($ billion)	1.5	100%	300.88 (117.18, 463.67)	156.02 (−35.21, 324.45)	401.00 (209.15, 563.02)
V2	threshold	NMB ($ billion)	1.7	20%	77.10 (−12.75, 163.34)	13.47 (−92.40, 97.95)	92.79 (−15.12, 191.20)
V2	threshold	NMB ($ billion)	1.7	50%	189.32 (24.86, 338.37)	39.88 (−153.00, 193.40)	230.76 (45.40, 401.40)
V2	threshold	NMB ($ billion)	1.7	80%	305.03 (136.52, 475.63)	87.23 (−107.73, 246.16)	369.74 (197.26, 543.65)
V2	threshold	NMB ($ billion)	1.7	100%	375.34 (218.51, 537.96)	110.57 (−92.29, 295.91)	449.87 (260.43, 602.46)
V2	threshold	NMB ($ billion)	2	20%	96.51 (−3.59, 199.44)	12.27 (−144.80, 149.40)	115.82 (−12.85, 241.25)
V2	threshold	NMB ($ billion)	2	50%	237.29 (38.48, 361.34)	27.86 (−224.24, 175.03)	278.55 (75.74, 417.55)
V2	threshold	NMB ($ billion)	2	80%	380.58 (189.93, 538.44)	59.57 (−195.98, 243.22)	441.14 (214.89, 603.82)
V2	threshold	NMB ($ billion)	2	100%	480.20 (251.39, 649.18)	82.60 (−163.77, 274.87)	547.65 (332.82, 702.43)
V2	threshold	NMB ($ billion)	3	20%	155.09 (48.09, 287.88)	4.20 (−164.87, 186.12)	173.53 (29.24, 323.30)
V2	threshold	NMB ($ billion)	3	50%	389.94 (183.64, 567.09)	10.28 (−247.85, 271.98)	430.46 (242.14, 621.07)
V2	threshold	NMB ($ billion)	3	80%	627.47 (413.89, 865.42)	16.61 (−257.12, 336.24)	673.68 (468.40, 958.89)
V2	threshold	NMB ($ billion)	3	100%	787.30 (556.13, 1068.43)	29.82 (−251.45, 364.75)	836.36 (601.25, 1070.03)
V2	threshold	NMB ($ billion)	5	20%	237.27 (79.70, 389.26)	−28.97 (−194.99, 122.52)	240.32 (67.46, 384.58)
V2	threshold	NMB ($ billion)	5	50%	589.59 (366.06, 828.63)	−56.53 (−363.41, 178.08)	599.76 (377.93, 858.01)
V2	threshold	NMB ($ billion)	5	80%	945.02 (670.93, 1275.73)	−69.84 (−402.22, 308.64)	968.79 (728.58, 1313.21)
V2	threshold	NMB ($ billion)	5	100%	1165.59 (892.37, 1477.99)	−98.65 (−442.35, 355.92)	1200.79 (904.45, 1531.25)
V2	threshold	Treatment courses used (million)	1.2	20%	6.54 (5.27, 7.74)	5.86 (4.28, 7.51)	5.86 (4.28, 7.51)
V2	threshold	Treatment courses used (million)	1.2	50%	16.54 (12.92, 19.21)	11.70 (7.68, 14.20)	11.78 (8.04, 14.20)
V2	threshold	Treatment courses used (million)	1.2	80%	26.53 (20.82, 30.38)	14.26 (8.27, 18.35)	14.39 (8.27, 18.48)
V2	threshold	Treatment courses used (million)	1.2	100%	33.34 (26.36, 38.02)	13.92 (7.15, 19.67)	14.12 (7.28, 19.90)
V2	threshold	Treatment courses used (million)	1.5	20%	11.85 (10.68, 13.25)	11.44 (10.31, 12.75)	11.45 (10.31, 12.75)

Scenarios	Infectiousness	Measures	R_t	Treatment %	Direct	Indirect	All (Direct + Indirect)
V2	threshold	Treatment courses used (million)	1.5	50%	29.60 (27.45, 32.52)	26.97 (24.91, 29.39)	27.01 (24.91, 29.65)
V2	threshold	Treatment courses used (million)	1.5	80%	47.43 (44.45, 50.94)	40.16 (36.44, 43.62)	40.27 (36.84, 43.72)
V2	threshold	Treatment courses used (million)	1.5	100%	59.45 (55.98, 63.39)	47.02 (42.44, 50.71)	47.17 (42.50, 51.10)
V2	threshold	Treatment courses used (million)	1.7	20%	13.92 (12.55, 15.45)	13.62 (12.09, 14.70)	13.63 (12.09, 14.70)
V2	threshold	Treatment courses used (million)	1.7	50%	34.81 (32.65, 37.66)	32.74 (30.48, 35.39)	32.80 (30.54, 35.39)
V2	threshold	Treatment courses used (million)	1.7	80%	55.73 (52.62, 58.88)	49.92 (46.92, 52.92)	50.02 (47.22, 53.01)
V2	threshold	Treatment courses used (million)	1.7	100%	69.79 (66.16, 73.24)	60.34 (57.30, 64.02)	60.40 (57.43, 64.22)
V2	threshold	Treatment courses used (million)	2	20%	16.45 (14.99, 18.42)	16.18 (14.86, 18.02)	16.19 (14.89, 18.02)
V2	threshold	Treatment courses used (million)	2	50%	41.30 (38.78, 43.95)	39.74 (37.53, 41.81)	39.76 (37.56, 41.94)
V2	threshold	Treatment courses used (million)	2	80%	66.08 (62.67, 69.13)	61.92 (58.58, 65.04)	62.01 (58.58, 65.14)
V2	threshold	Treatment courses used (million)	2	100%	82.73 (79.24, 87.15)	75.77 (72.26, 78.88)	75.87 (72.42, 79.14)
V2	threshold	Treatment courses used (million)	3	20%	24.45 (22.57, 26.85)	24.26 (22.40, 26.59)	24.25 (22.40, 26.72)
V2	threshold	Treatment courses used (million)	3	50%	61.22 (58.62, 64.05)	59.91 (56.97, 62.93)	59.95 (57.23, 62.90)
V2	threshold	Treatment courses used (million)	3	80%	97.90 (94.33, 100.92)	94.65 (91.40, 97.63)	94.77 (91.53, 97.99)
V2	threshold	Treatment courses used (million)	3	100%	122.75 (119.01, 126.23)	117.38 (113.87, 121.35)	117.55 (114.27, 121.42)
V2	threshold	Treatment courses used (million)	5	20%	37.66 (35.49, 39.90)	37.42 (35.29, 39.54)	37.45 (35.35, 39.54)
V2	threshold	Treatment courses used (million)	5	50%	94.27 (89.69, 98.19)	93.21 (88.80, 97.36)	93.29 (88.80, 97.43)
V2	threshold	Treatment courses used (million)	5	80%	150.84 (145.76, 155.22)	148.10 (142.96, 152.42)	148.31 (143.16, 152.49)
V2	threshold	Treatment courses used (million)	5	100%	188.93 (184.08, 193.51)	184.47 (180.10, 189.26)	184.75 (180.43, 189.32)
V2	threshold	Hospitalizations reduced (million)	1.2	20%	0.15 (−0.07, 0.36)	0.20 (−0.20, 0.56)	0.32 (0.03, 0.69)
V2	threshold	Hospitalizations reduced (million)	1.2	50%	0.37 (0.07, 0.69)	0.49 (0.00, 0.96)	0.74 (0.30, 1.15)
V2	threshold	Hospitalizations reduced (million)	1.2	80%	0.59 (0.20, 0.99)	0.77 (0.16, 1.25)	1.06 (0.49, 1.48)
V2	threshold	Hospitalizations reduced (million)	1.2	100%	0.72 (0.26, 1.25)	0.94 (0.36, 1.45)	1.22 (0.76, 1.75)
V2	threshold	Hospitalizations reduced (million)	1.5	20%	0.32 (−0.10, 0.66)	0.16 (−0.26, 0.63)	0.45 (0.10, 0.86)
V2	threshold	Hospitalizations reduced (million)	1.5	50%	0.79 (0.26, 1.35)	0.35 (−0.30, 0.99)	1.07 (0.56, 1.81)
V2	threshold	Hospitalizations reduced (million)	1.5	80%	1.26 (0.59, 1.98)	0.63 (−0.36, 1.55)	1.63 (0.76, 2.44)
V2	threshold	Hospitalizations reduced (million)	1.5	100%	1.55 (0.69, 2.34)	0.83 (−0.07, 1.65)	1.97 (1.05, 2.77)
V2	threshold	Hospitalizations reduced (million)	1.7	20%	0.40 (−0.03, 0.79)	0.10 (−0.43, 0.49)	0.47 (−0.03, 0.92)
V2	threshold	Hospitalizations reduced (million)	1.7	50%	0.97 (0.20, 1.65)	0.26 (−0.59, 0.96)	1.15 (0.33, 1.91)
V2	threshold	Hospitalizations reduced (million)	1.7	80%	1.56 (0.79, 2.31)	0.52 (−0.33, 1.25)	1.84 (1.05, 2.67)
V2	threshold	Hospitalizations reduced (million)	1.7	100%	1.92 (1.19, 2.70)	0.65 (−0.33, 1.45)	2.23 (1.35, 2.97)
V2	threshold	Hospitalizations reduced (million)	2	20%	0.49 (0.03, 0.92)	0.10 (−0.59, 0.69)	0.58 (0.00, 1.19)

Scenarios	Infectiousness	Measures	R_t	Treatment %	Direct	Indirect	All (Direct + Indirect)
V2	threshold	Hospitalizations reduced (million)	2	50%	1.20 (0.30, 1.81)	0.23 (−0.89, 0.89)	1.39 (0.43, 2.01)
V2	threshold	Hospitalizations reduced (million)	2	80%	1.93 (0.96, 2.67)	0.43 (−0.76, 1.28)	2.20 (1.12, 2.97)
V2	threshold	Hospitalizations reduced (million)	2	100%	2.43 (1.38, 3.23)	0.56 (−0.59, 1.42)	2.72 (1.75, 3.46)
V2	threshold	Hospitalizations reduced (million)	3	20%	0.78 (0.30, 1.38)	0.08 (−0.69, 0.92)	0.86 (0.23, 1.58)
V2	threshold	Hospitalizations reduced (million)	3	50%	1.95 (0.99, 2.73)	0.20 (−1.02, 1.38)	2.14 (1.22, 3.06)
V2	threshold	Hospitalizations reduced (million)	3	80%	3.14 (2.11, 4.18)	0.31 (−0.96, 1.78)	3.34 (2.31, 4.65)
V2	threshold	Hospitalizations reduced (million)	3	100%	3.94 (2.83, 5.27)	0.42 (−0.89, 1.91)	4.15 (3.00, 5.21)
V2	threshold	Hospitalizations reduced (million)	5	20%	1.18 (0.43, 1.91)	−0.06 (−0.79, 0.59)	1.20 (0.46, 1.88)
V2	threshold	Hospitalizations reduced (million)	5	50%	2.95 (1.94, 4.12)	−0.04 (−1.45, 1.09)	3.00 (1.98, 4.25)
V2	threshold	Hospitalizations reduced (million)	5	80%	4.73 (3.53, 6.26)	0.03 (−1.45, 1.71)	4.84 (3.59, 6.39)
V2	threshold	Hospitalizations reduced (million)	5	100%	5.85 (4.61, 7.45)	−0.01 (−1.58, 2.01)	6.00 (4.61, 7.58)
V3	sigmoid	Number of cases averted in U.S. (million)	1.2	20%	−0.12 (−3.00, 2.31)	10.82 (1.35, 22.47)	10.77 (1.35, 21.58)
V3	sigmoid	Number of cases averted in U.S. (million)	1.2	50%	−0.38 (−4.12, 2.90)	31.47 (15.75, 47.81)	31.14 (15.75, 47.08)
V3	sigmoid	Number of cases averted in U.S. (million)	1.2	80%	−0.59 (−5.47, 3.66)	50.12 (31.00, 70.18)	49.81 (30.94, 70.18)
V3	sigmoid	Number of cases averted in U.S. (million)	1.2	100%	−0.84 (−5.60, 3.39)	61.94 (44.68, 81.42)	61.51 (41.61, 81.38)
V3	sigmoid	Number of cases averted in U.S. (million)	1.5	20%	−0.17 (−2.08, 2.04)	5.91 (0.16, 12.03)	5.82 (0.16, 12.13)
V3	sigmoid	Number of cases averted in U.S. (million)	1.5	50%	−0.45 (−3.92, 2.08)	16.33 (7.41, 24.97)	16.11 (8.60, 24.91)
V3	sigmoid	Number of cases averted in U.S. (million)	1.5	80%	−0.73 (−4.09, 2.67)	27.39 (19.41, 36.94)	27.01 (18.29, 36.14)
V3	sigmoid	Number of cases averted in U.S. (million)	1.5	100%	−0.93 (−4.22, 2.21)	37.09 (29.09, 47.35)	36.61 (27.58, 46.39)
V3	sigmoid	Number of cases averted in U.S. (million)	1.7	20%	−0.19 (−1.98, 1.61)	5.19 (0.23, 9.59)	5.12 (0.13, 9.42)
V3	sigmoid	Number of cases averted in U.S. (million)	1.7	50%	−0.34 (−2.50, 2.21)	12.52 (6.56, 19.27)	12.30 (6.49, 18.81)
V3	sigmoid	Number of cases averted in U.S. (million)	1.7	80%	−0.65 (−3.43, 2.14)	21.64 (15.58, 28.01)	21.49 (15.29, 27.74)
V3	sigmoid	Number of cases averted in U.S. (million)	1.7	100%	−0.93 (−4.28, 2.04)	28.69 (22.90, 36.61)	28.28 (22.34, 36.08)
V3	sigmoid	Number of cases averted in U.S. (million)	2	20%	−0.17 (−2.57, 1.78)	3.56 (0.23, 6.46)	3.48 (0.03, 6.42)
V3	sigmoid	Number of cases averted in U.S. (million)	2	50%	−0.45 (−2.83, 1.68)	8.61 (4.48, 13.15)	8.48 (2.93, 12.98)
V3	sigmoid	Number of cases averted in U.S. (million)	2	80%	−0.75 (−3.39, 2.17)	15.37 (10.08, 21.22)	15.12 (9.82, 20.72)

Scenarios	Infectiousness	Measures	R_t	Treatment %	Direct	Indirect	All (Direct + Indirect)
V3	sigmoid	Number of cases averted in U.S. (million)	2	100%	−0.88 (−3.39, 2.21)	20.10 (14.20, 26.52)	19.77 (13.64, 26.13)
V3	sigmoid	Number of cases averted in U.S. (million)	3	20%	−0.07 (−0.43, 0.26)	0.76 (−0.07, 1.55)	0.75 (−0.07, 1.71)
V3	sigmoid	Number of cases averted in U.S. (million)	3	50%	−0.13 (−0.56, 0.49)	2.06 (1.15, 3.13)	2.00 (1.05, 3.10)
V3	sigmoid	Number of cases averted in U.S. (million)	3	80%	−0.16 (−0.69, 0.46)	3.41 (2.04, 4.68)	3.31 (1.98, 4.58)
V3	sigmoid	Number of cases averted in U.S. (million)	3	100%	−0.22 (−0.79, 0.43)	4.54 (2.90, 5.80)	4.40 (2.77, 5.86)
V3	sigmoid	Number of cases averted in U.S. (million)	5	20%	0.00 (−0.07, 0.07)	0.03 (−0.10, 0.13)	0.03 (−0.10, 0.13)
V3	sigmoid	Number of cases averted in U.S. (million)	5	50%	−0.01 (−0.10, 0.10)	0.08 (−0.10, 0.26)	0.08 (−0.10, 0.26)
V3	sigmoid	Number of cases averted in U.S. (million)	5	80%	−0.01 (−0.10, 0.10)	0.15 (−0.03, 0.36)	0.13 (−0.03, 0.36)
V3	sigmoid	Number of cases averted in U.S. (million)	5	100%	−0.01 (−0.13, 0.13)	0.20 (−0.03, 0.43)	0.18 (0.00, 0.40)
V3	sigmoid	Deaths reduced (thousand)	1.2	20%	13.83 (−10.76, 37.77)	16.68 (−25.08, 57.35)	31.17 (−10.81, 74.90)
V3	sigmoid	Deaths reduced (thousand)	1.2	50%	38.52 (−3.62, 89.16)	53.82 (−5.08, 116.02)	81.77 (34.03, 148.24)
V3	sigmoid	Deaths reduced (thousand)	1.2	80%	60.34 (5.43, 108.93)	86.51 (7.13, 157.01)	117.50 (55.24, 175.39)
V3	sigmoid	Deaths reduced (thousand)	1.2	100%	75.94 (11.17, 133.66)	107.09 (29.00, 174.19)	136.52 (69.56, 196.79)
V3	sigmoid	Deaths reduced (thousand)	1.5	20%	39.17 (−0.12, 81.56)	18.94 (−30.17, 85.48)	54.96 (5.33, 110.45)
V3	sigmoid	Deaths reduced (thousand)	1.5	50%	98.13 (12.68, 181.36)	47.02 (−53.91, 150.73)	133.24 (39.94, 210.32)
V3	sigmoid	Deaths reduced (thousand)	1.5	80%	157.20 (53.38, 243.62)	68.36 (−27.07, 170.86)	196.67 (106.07, 293.55)
V3	sigmoid	Deaths reduced (thousand)	1.5	100%	191.94 (113.41, 274.38)	97.48 (−12.74, 213.41)	241.77 (166.98, 332.90)
V3	sigmoid	Deaths reduced (thousand)	1.7	20%	46.11 (−8.66, 94.07)	12.65 (−62.72, 74.95)	57.53 (−14.22, 132.01)
V3	sigmoid	Deaths reduced (thousand)	1.7	50%	113.71 (21.24, 201.09)	36.38 (−69.50, 139.28)	145.77 (44.69, 242.28)
V3	sigmoid	Deaths reduced (thousand)	1.7	80%	184.69 (78.06, 275.95)	60.59 (−52.71, 186.03)	223.39 (123.57, 335.84)
V3	sigmoid	Deaths reduced (thousand)	1.7	100%	231.30 (112.47, 348.62)	84.64 (−37.65, 212.72)	274.23 (165.21, 388.33)
V3	sigmoid	Deaths reduced (thousand)	2	20%	55.95 (−12.04, 112.31)	10.74 (−87.13, 80.44)	69.07 (−12.40, 144.24)
V3	sigmoid	Deaths reduced (thousand)	2	50%	145.65 (22.98, 253.46)	28.23 (−80.26, 142.57)	172.26 (55.27, 269.35)
V3	sigmoid	Deaths reduced (thousand)	2	80%	236.46 (78.41, 355.13)	55.79 (−58.47, 154.98)	271.54 (152.20, 367.46)
V3	sigmoid	Deaths reduced (thousand)	2	100%	297.70 (125.48, 429.83)	72.75 (−61.56, 184.70)	334.87 (189.30, 437.30)
V3	sigmoid	Deaths reduced (thousand)	3	20%	99.55 (37.83, 177.92)	11.38 (−88.92, 121.61)	108.96 (21.69, 200.82)

Scenarios	Infectiousness	Measures	R_t	Treatment %	Direct	Indirect	All (Direct + Indirect)
V3	sigmoid	Deaths reduced (thousand)	3	50%	253.66 (121.44, 374.01)	28.16 (−129.14, 135.85)	271.04 (149.96, 389.81)
V3	sigmoid	Deaths reduced (thousand)	3	80%	401.91 (276.73, 511.36)	41.95 (−143.92, 174.49)	425.44 (301.54, 559.92)
V3	sigmoid	Deaths reduced (thousand)	3	100%	504.77 (371.43, 640.87)	50.28 (−128.64, 185.57)	525.93 (395.51, 672.69)
V3	sigmoid	Deaths reduced (thousand)	5	20%	151.95 (63.42, 229.30)	0.90 (−99.91, 97.17)	156.10 (55.43, 233.34)
V3	sigmoid	Deaths reduced (thousand)	5	50%	377.19 (252.90, 510.31)	−1.17 (−157.28, 149.66)	383.18 (234.69, 513.12)
V3	sigmoid	Deaths reduced (thousand)	5	80%	597.26 (423.69, 769.79)	5.57 (−205.87, 226.22)	613.42 (449.77, 791.40)
V3	sigmoid	Deaths reduced (thousand)	5	100%	743.34 (578.10, 990.40)	1.95 (−219.56, 271.67)	760.42 (581.84, 998.20)
V3	sigmoid	NMB ($ billion)	1.2	20%	21.52 (−23.43, 67.77)	27.51 (−49.79, 103.60)	53.78 (−24.03, 125.87)
V3	sigmoid	NMB ($ billion)	1.2	50%	60.33 (−13.57, 136.17)	91.21 (−21.58, 210.19)	141.62 (47.05, 268.21)
V3	sigmoid	NMB ($ billion)	1.2	80%	95.11 (−2.17, 171.22)	149.38 (23.41, 277.38)	205.69 (94.70, 307.31)
V3	sigmoid	NMB ($ billion)	1.2	100%	119.68 (−0.80, 230.59)	187.07 (54.82, 300.88)	241.02 (125.68, 348.97)
V3	sigmoid	NMB ($ billion)	1.5	20%	62.30 (−11.80, 136.89)	25.88 (−57.61, 129.27)	89.06 (6.69, 177.41)
V3	sigmoid	NMB ($ billion)	1.5	50%	154.44 (11.93, 277.73)	63.05 (−113.31, 218.76)	215.29 (59.40, 337.65)
V3	sigmoid	NMB ($ billion)	1.5	80%	248.48 (64.16, 379.89)	93.93 (−81.19, 235.40)	320.12 (172.36, 469.64)
V3	sigmoid	NMB ($ billion)	1.5	100%	303.61 (136.79, 437.05)	141.46 (−63.87, 319.62)	396.05 (242.23, 544.14)
V3	sigmoid	NMB ($ billion)	1.7	20%	73.64 (−10.68, 153.90)	13.57 (−133.00, 128.02)	92.01 (−20.11, 207.94)
V3	sigmoid	NMB ($ billion)	1.7	50%	178.30 (30.45, 300.87)	40.38 (−161.57, 225.27)	231.93 (41.43, 396.19)
V3	sigmoid	NMB ($ billion)	1.7	80%	291.57 (83.02, 451.51)	74.23 (−131.66, 283.05)	358.74 (172.34, 556.19)
V3	sigmoid	NMB ($ billion)	1.7	100%	365.30 (172.03, 544.61)	111.40 (−107.46, 360.48)	443.11 (245.86, 624.27)
V3	sigmoid	NMB ($ billion)	2	20%	87.12 (−16.38, 182.30)	9.39 (−138.67, 143.49)	110.38 (−23.31, 229.11)
V3	sigmoid	NMB ($ billion)	2	50%	227.99 (21.34, 413.35)	25.92 (−172.69, 216.54)	273.66 (75.75, 444.19)
V3	sigmoid	NMB ($ billion)	2	80%	373.70 (112.23, 580.56)	62.86 (−135.78, 245.16)	434.97 (236.08, 600.06)
V3	sigmoid	NMB ($ billion)	2	100%	471.37 (177.97, 702.54)	84.40 (−179.88, 292.65)	537.26 (287.70, 727.58)
V3	sigmoid	NMB ($ billion)	3	20%	154.17 (51.52, 273.51)	3.51 (−158.82, 185.08)	168.75 (32.52, 321.46)

Scenarios	Infectiousness	Measures	R_t	Treatment %	Direct	Indirect	All (Direct + Indirect)
V3	sigmoid	NMB ($ billion)	3	50%	396.63 (177.90, 586.99)	16.70 (−206.54, 207.29)	426.45 (230.79, 603.19)
V3	sigmoid	NMB ($ billion)	3	80%	628.62 (425.56, 807.12)	23.58 (−254.36, 267.38)	668.95 (437.00, 863.34)
V3	sigmoid	NMB ($ billion)	3	100%	787.60 (534.26, 1020.35)	25.19 (−258.68, 298.63)	826.38 (584.43, 1049.06)
V3	sigmoid	NMB ($ billion)	5	20%	231.36 (82.93, 359.40)	−23.49 (−198.21, 135.26)	239.20 (77.17, 368.28)
V3	sigmoid	NMB ($ billion)	5	50%	579.66 (347.04, 803.20)	−53.14 (−342.45, 207.84)	592.66 (358.26, 802.81)
V3	sigmoid	NMB ($ billion)	5	80%	922.76 (653.13, 1223.77)	−72.10 (−450.78, 317.79)	951.38 (660.49, 1266.58)
V3	sigmoid	NMB ($ billion)	5	100%	1147.35 (891.51, 1540.52)	−97.25 (−450.15, 352.45)	1179.06 (893.80, 1582.14)
V3	sigmoid	Treatment courses used (million)	1.2	20%	6.38 (4.91, 7.61)	5.68 (4.15, 6.99)	5.68 (4.15, 6.99)
V3	sigmoid	Treatment courses used (million)	1.2	50%	16.08 (12.22, 18.71)	11.07 (7.28, 14.30)	11.12 (7.35, 14.30)
V3	sigmoid	Treatment courses used (million)	1.2	80%	25.77 (20.69, 29.95)	12.93 (7.25, 17.66)	13.00 (7.25, 17.69)
V3	sigmoid	Treatment courses used (million)	1.2	100%	32.36 (26.00, 36.87)	12.37 (6.66, 17.59)	12.51 (6.66, 18.22)
V3	sigmoid	Treatment courses used (million)	1.5	20%	11.93 (10.68, 13.31)	11.55 (10.38, 12.75)	11.56 (10.38, 12.75)
V3	sigmoid	Treatment courses used (million)	1.5	50%	29.79 (27.31, 32.32)	27.33 (24.78, 29.59)	27.37 (24.78, 29.65)
V3	sigmoid	Treatment courses used (million)	1.5	80%	47.75 (44.61, 50.77)	40.73 (37.59, 44.02)	40.85 (37.76, 44.15)
V3	sigmoid	Treatment courses used (million)	1.5	100%	59.79 (56.31, 63.52)	47.94 (42.80, 52.22)	48.08 (43.62, 52.52)
V3	sigmoid	Treatment courses used (million)	1.7	20%	13.68 (12.45, 15.29)	13.36 (12.06, 14.76)	13.36 (12.06, 14.76)
V3	sigmoid	Treatment courses used (million)	1.7	50%	33.99 (31.66, 35.91)	32.00 (29.13, 34.17)	32.02 (29.06, 34.17)
V3	sigmoid	Treatment courses used (million)	1.7	80%	54.53 (51.04, 57.03)	48.92 (45.67, 51.53)	48.95 (45.70, 51.47)
V3	sigmoid	Treatment courses used (million)	1.7	100%	68.34 (64.61, 70.94)	58.98 (55.91, 62.17)	59.09 (56.01, 62.01)
V3	sigmoid	Treatment courses used (million)	2	20%	16.09 (14.53, 17.66)	15.86 (14.53, 17.40)	15.87 (14.53, 17.36)
V3	sigmoid	Treatment courses used (million)	2	50%	40.42 (38.19, 42.50)	38.81 (36.47, 41.35)	38.83 (36.70, 41.45)
V3	sigmoid	Treatment courses used (million)	2	80%	64.80 (61.78, 68.14)	60.24 (56.94, 63.69)	60.33 (57.26, 63.72)
V3	sigmoid	Treatment courses used (million)	2	100%	81.08 (77.26, 84.71)	73.87 (70.18, 77.43)	73.95 (70.35, 77.96)
V3	sigmoid	Treatment courses used (million)	3	20%	24.34 (22.27, 26.36)	24.14 (22.21, 26.52)	24.14 (22.21, 26.56)
V3	sigmoid	Treatment courses used (million)	3	50%	60.99 (58.15, 63.85)	59.64 (56.61, 62.73)	59.67 (56.80, 62.60)
V3	sigmoid	Treatment courses used (million)	3	80%	97.51 (94.33, 101.12)	94.16 (91.20, 97.03)	94.21 (91.07, 97.07)
V3	sigmoid	Treatment courses used (million)	3	100%	121.98 (119.11, 125.50)	116.64 (113.31, 120.10)	116.79 (113.24, 120.10)
V3	sigmoid	Treatment courses used (million)	5	20%	37.56 (34.99, 39.77)	37.36 (34.86, 39.74)	37.38 (34.96, 39.83)
V3	sigmoid	Treatment courses used (million)	5	50%	93.85 (89.78, 97.89)	92.61 (88.20, 96.87)	92.72 (88.50, 96.84)

Scenarios	Infectiousness	Measures	R_t	Treatment %	Direct	Indirect	All (Direct + Indirect)
V3	sigmoid	Treatment courses used (million)	5	80%	150.43 (145.30, 154.89)	147.35 (142.17, 151.96)	147.54 (142.83, 152.26)
V3	sigmoid	Treatment courses used (million)	5	100%	188.39 (183.56, 192.95)	183.51 (178.38, 187.81)	183.76 (178.88, 188.10)
V3	sigmoid	Hospitalizations reduced (million)	1.2	20%	0.12 (−0.10, 0.33)	0.14 (−0.23, 0.49)	0.27 (−0.07, 0.59)
V3	sigmoid	Hospitalizations reduced (million)	1.2	50%	0.33 (−0.03, 0.72)	0.46 (−0.07, 1.02)	0.69 (0.26, 1.25)
V3	sigmoid	Hospitalizations reduced (million)	1.2	80%	0.52 (0.03, 0.89)	0.73 (0.16, 1.32)	0.99 (0.46, 1.45)
V3	sigmoid	Hospitalizations reduced (million)	1.2	100%	0.65 (0.07, 1.19)	0.90 (0.30, 1.48)	1.16 (0.59, 1.71)
V3	sigmoid	Hospitalizations reduced (million)	1.5	20%	0.32 (−0.03, 0.66)	0.15 (−0.26, 0.63)	0.44 (0.03, 0.86)
V3	sigmoid	Hospitalizations reduced (million)	1.5	50%	0.79 (0.16, 1.32)	0.35 (−0.43, 1.05)	1.06 (0.36, 1.61)
V3	sigmoid	Hospitalizations reduced (million)	1.5	80%	1.27 (0.40, 1.88)	0.52 (−0.30, 1.22)	1.58 (0.89, 2.31)
V3	sigmoid	Hospitalizations reduced (million)	1.5	100%	1.56 (0.76, 2.24)	0.76 (−0.23, 1.55)	1.95 (1.19, 2.70)
V3	sigmoid	Hospitalizations reduced (million)	1.7	20%	0.38 (0.00, 0.76)	0.09 (−0.63, 0.63)	0.46 (−0.07, 1.02)
V3	sigmoid	Hospitalizations reduced (million)	1.7	50%	0.91 (0.26, 1.48)	0.26 (−0.66, 1.09)	1.15 (0.30, 1.94)
V3	sigmoid	Hospitalizations reduced (million)	1.7	80%	1.49 (0.49, 2.17)	0.46 (−0.49, 1.38)	1.78 (0.89, 2.64)
V3	sigmoid	Hospitalizations reduced (million)	1.7	100%	1.86 (0.99, 2.67)	0.65 (−0.36, 1.75)	2.20 (1.22, 3.00)
V3	sigmoid	Hospitalizations reduced (million)	2	20%	0.45 (−0.03, 0.89)	0.09 (−0.59, 0.72)	0.55 (−0.07, 1.09)
V3	sigmoid	Hospitalizations reduced (million)	2	50%	1.16 (0.23, 2.04)	0.21 (−0.72, 1.02)	1.36 (0.43, 2.14)
V3	sigmoid	Hospitalizations reduced (million)	2	80%	1.89 (0.76, 2.87)	0.44 (−0.46, 1.25)	2.16 (1.22, 2.90)
V3	sigmoid	Hospitalizations reduced (million)	2	100%	2.39 (1.09, 3.46)	0.57 (−0.72, 1.52)	2.67 (1.55, 3.59)
V3	sigmoid	Hospitalizations reduced (million)	3	20%	0.77 (0.33, 1.28)	0.07 (−0.66, 0.92)	0.84 (0.26, 1.48)
V3	sigmoid	Hospitalizations reduced (million)	3	50%	1.98 (0.99, 2.83)	0.23 (−0.86, 1.09)	2.12 (1.22, 2.93)
V3	sigmoid	Hospitalizations reduced (million)	3	80%	3.15 (2.24, 4.02)	0.35 (−0.96, 1.28)	3.32 (2.31, 4.22)
V3	sigmoid	Hospitalizations reduced (million)	3	100%	3.94 (2.67, 5.01)	0.40 (−0.86, 1.58)	4.10 (2.97, 5.14)
V3	sigmoid	Hospitalizations reduced (million)	5	20%	1.15 (0.40, 1.78)	−0.03 (−0.86, 0.72)	1.19 (0.46, 1.85)
V3	sigmoid	Hospitalizations reduced (million)	5	50%	2.91 (1.91, 3.92)	−0.02 (−1.32, 1.22)	2.96 (1.88, 3.95)
V3	sigmoid	Hospitalizations reduced (million)	5	80%	4.63 (3.43, 6.10)	0.02 (−1.78, 1.88)	4.75 (3.43, 6.19)
V3	sigmoid	Hospitalizations reduced (million)	5	100%	5.76 (4.51, 7.64)	0.00 (−1.68, 1.98)	5.90 (4.55, 7.71)

References

1. Hernandez-Vargas EA, Velasco-Hernandez JX. In-host modelling of COVID-19 in humans. Annu Rev Control. 2020;50:448–56. **PMID 33020692**

2. Czuppon P, Débarre F, Gonçalves A, Tenaillon O, Perelson AS, Guedj J, et al. Success of prophylactic antiviral therapy for SARS-CoV-2: predicted critical efficacies and impact of different drug-specific mechanisms of action. PLOS Comput Biol. 2021;17:e1008752. PubMed https://doi.org/10.1371/journal.pcbi.1008752

3. Backer JA, Klinkenberg D, Wallinga J. Incubation period of 2019 novel coronavirus (2019-nCoV) infections among travellers from Wuhan, China, 20–28 January 2020. Euro Surveill. 2020;25:2000062. PubMed https://doi.org/10.2807/1560-7917.ES.2020.25.5.2000062

4. Hammond J, Leister-Tebbe H, Gardner A, Abreu P, Bao W, Wisemandle W, et al.; EPIC-HR Investigators. Oral nirmatrelvir for high-risk, nonhospitalized adults with Covid-19. N Engl J Med. 2022;386:1397–408. PubMed https://doi.org/10.1056/NEJMoa2118542

5. Handel A, Longini IM Jr, Antia R. Neuraminidase inhibitor resistance in influenza: assessing the danger of its generation and spread. PLOS Comput Biol. 2007;3:e240. PubMed https://doi.org/10.1371/journal.pcbi.0030240

6. Traynard P, Ayral G, Twarogowska M, Chauvin J. Efficient pharmacokinetic modeling workflow with the MonolixSuite: a case study of remifentanil. CPT Pharmacometrics Syst Pharmacol. 2020;9:198–210. PubMed https://doi.org/10.1002/psp4.12500

7. Miao H, Xia X, Perelson AS, Wu H. On identifiability of nonlinear ode models and applications in viral dynamics. SIAM Rev Soc Ind Appl Math. 2011;53:3–39. PubMed https://doi.org/10.1137/090757009

8. Wölfel R, Corman VM, Guggemos W, Seilmaier M, Zange S, Müller MA, et al. Virological assessment of hospitalized patients with COVID-2019. Nature. 2020;581:465–9. PubMed https://doi.org/10.1038/s41586-020-2196-x

9. Grijalva CG, Rolfes MA, Zhu Y, McLean HQ, Hanson KE, Belongia EA, et al. Transmission of SARS-CoV-2 infections in households—Tennessee and Wisconsin, April–September 2020. MMWR Morb Mortal Wkly Rep. 2020;69:1631–4. PubMed

10. Mathieu E, Ritchie H, Rodés-Guirao L, Appel C, Giattino C, Hasell J, et al. Coronavirus pandemic (COVID-19). 2022 Dec 8 [cited 2022 Nov 19]. https://ourworldindata.org/coronavirus

11. U.S. Food and Drug Administration. FDA authorizes Pfizer-BioNTech COVID-19 Vaccine for emergency use in children 5 through 11 years of age [cited 2022 Dec 14]. https://www.fda.gov/news-events/press-announcements/fda-authorizes-pfizer-biontech-covid-19-vaccine-emergency-use-children-5-through-11-years-age

12. U.S. Food and Drug Administration. Coronavirus (COVID-19) update: FDA authorizes Pfizer-BioNTech COVID-19 Vaccine for emergency use in adolescents in another important action in fight against pandemic [cited 2023 Jan 20]. https://www.fda.gov/news-events/press-announcements/coronavirus-covid-19-update-fda-authorizes-pfizer-biontech-covid-19-vaccine-emergency-use

13. Centers for Disease Control and Prevention. Joint statement from HHS public health and medical experts on COVID-19 booster shots [cited 2023 Jan 20]. https://www.cdc.gov/media/releases/2021/s0818-covid-19-booster-shots.html

14. Centers for Disease Control and Prevention. CDC statement on ACIP booster recommendations [cited 2022 Dec 15]. https://www.cdc.gov/media/releases/2021/p0924-booster-recommendations-.html

15. Centers for Disease Control and Prevention. CDC recommends Pfizer booster at 5 months, additional primary dose for certain immunocompromised children [cited 2022 Dec 15]. https://www.cdc.gov/media/releases/2022/s0104-Pfizer-Booster.html

16. Centers for Disease Control and Prevention. COVIDVaxView [cited 2022 Mar 12]. https://www.cdc.gov/vaccines/imz-managers/coverage/covidvaxview/index.html

17. Du Z, Pandey A, Bai Y, Fitzpatrick M, Chinazzi M, Pastore y Piontti A, et al. Comparative cost-effectiveness of SARS-CoV-2 testing strategies in the USA: a modelling study. Lancet Public Health. 2021;6:e184–91. **PMID 33549196**

18. Shah MM, Joyce B, Plumb ID, Sahakian S, Feldstein LR, Barkley E, et al. Paxlovid associated with decreased hospitalization rate among adults with COVID-19—United States, April–September 2022. MMWR Morb Mortal Wkly Rep. 2022;71:1531–7. PubMed https://doi.org/10.15585/mmwr.mm7148e2

19. U.S. Federal Highway Administration. 2017 national household travel survey [cited 2020 Jun 16]. https://nhts.ornl.gov/

20. Mistry D, Litvinova M, Pastore Y Piontti A, Chinazzi M, Fumanelli L, Gomes MFC, et al. Inferring high-resolution human mixing patterns for disease modeling. Nat Commun. 2021;12:323. PubMed https://doi.org/10.1038/s41467-020-20544-y

21. US Census Bureau. National population by characteristics: 2010–2019 [cited 2020 Oct 1]. https://www.census.gov/data/tables/time-series/demo/popest/2010s-national-detail.html

22. Mathieu E, Ritchie H, Rodés-Guirao L, Appel C, Giattino C, Hasell J, et al. Coronavirus pandemic (COVID-19). 2020 Mar 5 [cited 2023 Aug 28]. https://ourworldindata.org/covid-cases

23. Centers for Disease Control and Prevention. Estimated COVID-19 burden [cited 2023 Aug 30]. https://www.cdc.gov/coronavirus/2019-ncov/cases-updates/burden.html

24. Centers for Disease Control and Prevention. Estimated COVID-19 burden [cited 2022 Nov 19]. https://www.cdc.gov/coronavirus/2019-ncov/cases-updates/burden.html

25. Ferretti L, Wymant C, Kendall M, Zhao L, Nurtay A, Abeler-Dörner L, et al. Quantifying SARS-CoV-2 transmission suggests epidemic control with digital contact tracing. Science. 2020;368:eabb6936. PubMed https://doi.org/10.1126/science.abb6936

26. He X, Lau EHY, Wu P, Deng X, Wang J, Hao X, et al. Temporal dynamics in viral shedding and transmissibility of COVID-19. Nat Med. 2020;26:672–5. PubMed https://doi.org/10.1038/s41591-020-0869-5

27. Aleta A, Martín-Corral D, Pastore Y Piontti A, Ajelli M, Litvinova M, Chinazzi M, et al. Modelling the impact of testing, contact tracing and household quarantine on second waves of COVID-19. Nat Hum Behav. 2020;4:964–71. PubMed https://doi.org/10.1038/s41562-020-0931-9

28. Verity R, Okell LC, Dorigatti I, Winskill P, Whittaker C, Imai N, et al. Estimates of the severity of coronavirus disease 2019: a model-based analysis. Lancet Infect Dis. 2020;20:669–77. PubMed https://doi.org/10.1016/S1473-3099(20)30243-7

29. Tindale LC, Stockdale JE, Coombe M, Garlock ES, Lau WYV, Saraswat M, et al. Evidence for transmission of COVID-19 prior to symptom onset. Elife. 2020;9:e57419. **PMID 32568070**

30. Adjei S, Hong K, Molinari NM, Bull-Otterson L, Ajani UA, Gundlapalli AV, et al. Mortality risk among patients hospitalized primarily for COVID-19 during the Omicron and Delta variant pandemic periods—United States, April 2020–June 2022. MMWR Morb Mortal Wkly Rep. 2022;71:1182–9. PubMed https://doi.org/10.15585/mmwr.mm7137a4

31. Wang X, Pasco RF, Du Z, Petty M, Fox SJ, Galvani AP, et al. Impact of social distancing measures on coronavirus disease healthcare demand, Central Texas, USA. Emerg Infect Dis. 2020;26:2361–9. PubMed https://doi.org/10.3201/eid2610.201702

32. Arias E, Heron M, Xu J. United States life tables, 2014. Natl Vital Stat Rep. 2017;66:1–64. PubMed

33. Andrews N, Stowe J, Kirsebom F, Toffa S, Rickeard T, Gallagher E, et al. Covid-19 vaccine effectiveness against the Omicron (B.1.1.529) variant. N Engl J Med. 2022;386:1532–46. PubMed https://doi.org/10.1056/NEJMoa2119451

34. Dagan N, Barda N, Kepten E, Miron O, Perchik S, Katz MA, et al. BNT162b2 mRNA Covid-19 vaccine in a nationwide mass vaccination setting. N Engl J Med. 2021;384:1412–23. PubMed https://doi.org/10.1056/NEJMoa2101765

35. Tang P, Hasan MR, Chemaitelly H, Yassine HM, Benslimane FM, Al Khatib HA, et al. BNT162b2 and mRNA-1273 COVID-19 vaccine effectiveness against the SARS-CoV-2 Delta variant in Qatar. Nat Med. 2021;27:2136–43. PubMed https://doi.org/10.1038/s41591-021-01583-4

36. Andrews N, Tessier E, Stowe J, Gower C, Kirsebom F, Simmons R, et al. Duration of protection against mild and severe disease by Covid-19 vaccines. N Engl J Med. 2022;386:340–50. PubMed https://doi.org/10.1056/NEJMoa2115481

37. Hansen CH, Michlmayr D, Gubbels SM, Mølbak K, Ethelberg S. Assessment of protection against reinfection with SARS-CoV-2 among 4 million PCR-tested individuals in Denmark in 2020: a population-level observational study. Lancet. 2021;397:1204–12. PubMed https://doi.org/10.1016/S0140-6736(21)00575-4

38. Pouwels KB, Pritchard E, Matthews PC, Stoesser N, Eyre DW, Vihta KD, et al. Effect of Delta variant on viral burden and vaccine effectiveness against new SARS-CoV-2 infections in the UK. Nat Med. 2021;27:2127–35. PubMed https://doi.org/10.1038/s41591-021-01548-7

39. Chemaitelly H, Ayoub HH, AlMukdad S, Coyle P, Tang P, Yassine HM, et al. Protection from previous natural infection compared with mRNA vaccination against SARS-CoV-2 infection and severe COVID-19 in Qatar: a retrospective cohort study. Lancet Microbe. 2022;3:e944–55. PubMed https://doi.org/10.1016/S2666-5247(22)00287-7

40. Robbins R, Zimmer CFDA. Clears Pfizer's Covid Pill for High-Risk Patients 12 and Older. 2021 Dec 22 [cited 2022 Apr 29]. https://www.nytimes.com/2021/12/22/health/pfizer-covid-pill-fda-paxlovid.html

41. FAIR Health, Inc. Key characteristics of COVID-19 patients: profiles based on analysis of private healthcare claims [cited 2020 Nov 28]. https://s3.amazonaws.com/media2.fairhealth.org/brief/asset/Key%20Characteristics%20of%20COVID-19%20Patients%20-

%20Profiles%20Based%20on%20Analysis%20of%20Private%20Healthcare%20Claims%20-%20A%20FAIR%20Health%20Brief.pdf

42. Larremore DB, Wilder B, Lester E, Shehata S, Burke JM, Hay JA, et al. Test sensitivity is secondary to frequency and turnaround time for COVID-19 screening. Sci Adv. 2021;7:eabd5393. PubMed https://doi.org/10.1126/sciadv.abd5393

43. Jones TC, Biele G, Mühlemann B, Veith T, Schneider J, Beheim-Schwarzbach J, et al. Estimating infectiousness throughout SARS-CoV-2 infection course. Science. 2021;373:eabi5273. PubMed https://doi.org/10.1126/science.abi5273
